# Tetrahydromagnolol induces autophagic cell death by targeting the m^6^A reader protein YTHDF2 and enhances the efficacy of anti-PD-1 immunotherapy in pancreatic cancer cells

**DOI:** 10.7150/thno.112612

**Published:** 2025-07-02

**Authors:** Guohua Li, Qibiao Wu, Yiping Mou, Yunhao Qiao, Lijun Jin, Qian Shi, Ruonan Zhang, Jie Li, Yitian Sun, Aili Zhang, Haiyang Jiang, Zijing Yang, Zhiyu Zhu, Mengmeng Ma, Xiaoyu Sun, Xinbing Sui

**Affiliations:** 1 School of Pharmacy, Hangzhou Normal University, Hangzhou, Zhejiang, China.; 2 State Key Laboratory of Quality Research in Chinese Medicines, Faculty of Chinese Medicine, Macau University of Science and Technology, Macau, P.R. China.; 3 Department of Gastrointestinal and Pancreatic Surgery, Zhejiang Provincial People's Hospital, People's Hospital of Hangzhou Medical College, Hangzhou, Zhejiang, China.; 4 Department of Traditional Chinese Medicine, The Second Affiliated Hospital, School of Medicine, Zhejiang University, Hangzhou, Zhejiang, China.

**Keywords:** Tetrahydromagnolol, pancreatic cancer, autophagic cell death, YTHDF2, PD-1.

## Abstract

**Rationale:** Tetrahydromagnolol (THM) is a bioactive compound derived from *Magnolia officinalis*. Although other compounds from this plant, such as magnolol and honokiol, have shown significant anticancer potential, the anticancer activities of THM remain unreported. This study aims to investigate the anticancer effects and underlying molecular mechanisms of THM in pancreatic cancer cells.

**Methods:** In this study, the effects of THM on pancreatic cancer cells were investigated by various experiments both *in vitro* and *in vivo*. The molecular target of THM in pancreatic cancer cells was determined by transcriptomics, ligand coupled epoxy-activated magnetic beads, CETSA, SPR analysis, ITC analysis, LC-MS/MS analysis, and MD simulations.

**Results:** Our findings reveal that THM significantly suppresses pancreatic cancer cell proliferation and induces cell death. Autophagic cell death is demonstrated to predominantly contribute to THM-triggered cell death. Importantly, YTHDF2, the m^6^A reader protein, is identified as a direct anticancer target of THM. Further investigations have shown that THM binds to YTHDF2, blocking its ability to recognize m^6^A modifications on the autophagy-related gene mRNAs *ATG5* and *ATG7*. Notably, a medium dose of THM exhibits anticancer efficacy comparable to gemcitabine (GEM), the first-line treatment for pancreatic cancer, and the high dose of THM showing superior anticancer effects than GEM treatment. Moreover, THM enhances the efficacy of anti-PD-1 immunotherapy in pancreatic cancer models.

**Conclusions:** This study presents the first evidence that THM promotes cell death in pancreatic cancer cells by inducing autophagy and YTHDF2 is identified as a direct binding target of THM. Targeting YTHDF2 is a critical determiner for THM-induced autophagic cell death and the immunosensitizing effect of THM with anti-PD-1 inhibitor in pancreatic cancer. Therefore, THM may function as a candidate anticancer drug for pancreatic cancer treatment, either alone or in combination with anti-PD-1 immunotherapy.

## Introduction

Pancreatic cancer is a highly aggressive malignant tumor with late diagnosis and very poor prognosis worldwide 1. Surgery remains the most effective treatment for pancreatic cancer, but most cases are diagnosed at an advanced stage when surgery is no longer a viable option 2. To date, chemotherapy persists as the established therapeutic regimen for patients battling advanced pancreatic cancer. Nonetheless, its toxicity and therapeutic resistance limit their clinical application 3-5. Therefore, it is urgent to identify new anticancer drugs for pancreatic cancer treatment. Currently, natural products for cancer prevention and treatment are attracting increasing attention. A growing number of natural compounds have been demonstrated to exert bioactive beneficial impacts on diverse cancer types, including pancreatic cancer 6-9.

The bark of *Magnolia officinalis* functions as a traditional herbal remedy, widely utilized in in China, Korea, Japan and other Asian countries 10. Previous studies have reported that natural bioactive compounds of *Magnolia officinalis* exhibit diverse biological activities, including anti-tumor, antibacterial, analgesic, anti-inflammatory, and anti-oxidative properties 11. Tetrahydromagnolol (THM) is a natural small molecule compound derived from *Magnolia officinalis* that has been documented to function as an effective selective agonist of the cannabinoid receptor CB2, which might influence the hypoalgesia associated with inflammation 12, but its anticancer potential remains to be elucidated.

N6-methyladenosine (m^6^A), the foremost internal modification within eukaryotic mRNA, participates in modulating gene expression and a spectrum of biological processes 13. YTH N6-methyladenosine RNA binding protein F2 (YTHDF2), a pivotal reader protein for m^6^A modification reader protein, selectively recognizes RNAs bound to m^6^A-modified RNAs, leading to mRNA degradation 14. Substantial evidence has revealed that YTHDF2 is intimately associated with tumor cell proliferation and metastasis, and its pivotal role in the post-transcriptional regulation of pancreatic cancer 15-17. Therefore, targeting YTHDF2 may be a highly promising approach for pancreatic cancer treatment.

Autophagy is a multistep-regulated cellular degradation process: double-membrane autophagosomes engulf cellular components, which then fuse with lysosomal compartments containing organelles and protein, are degraded 18, 19. Increasing evidence has indicated that m^6^A modification participates in in the autophagic process 20. It has been reported that the key autophagy-related genes *ATG5* and *ATG7* can be recognized by the m^6^A-reader protein YTHDF2, resulting in the degradation of their mRNAs, thereby reducing autophagy 21. In addition, autophagic cell death, a form of non-apoptotic regulated cell death (RCD), enhances the immunogenicity of tumor cells and promotes antitumor immunity 22. As a result, autophagic cell death inducers are utilized alongside anti-PD-1 (αPD-1) immunotherapy to inhibit tumor growth.

Our study investigated the effect of the natural compound THM on pancreatic cancer cells. The results revealed that THM exerted its anticancer effect by inducing autophagic cell death. Mechanistically, we found that THM can directly target YTHDF2, thereby blocking the reading of m^6^A modifications on autophagy-associated genes *ATG5* and *ATG7*. Further studies showed that the therapeutic effect of a medium dose of THM was comparable to that of the treatment with gemcitabine (GEM), and that the high dose of THM had superior anticancer activity than GEM. Notably, THM was demonstrated to increase the therapeutic efficacy of αPD-1 immunotherapy in the orthotopic Pan02-luc model. Taken together, our findings showed, for the first time, that YTHDF2 is a direct binding target of THM and targeting YTHDF2 is a crucial factor in THM-induced autophagic cell death. Additionally, THM was shown to improve the effectiveness of αPD-1 immunotherapy against pancreatic cancer. Thus, THM could be developed into a new candidate anticancer drug to be used alone or in combination with αPD-1 for treating pancreatic cancer patients.

## Results

### THM reduced cell viability, suppressed cell proliferation and triggered cell death in pancreatic cancer cells

**Figure 1:**
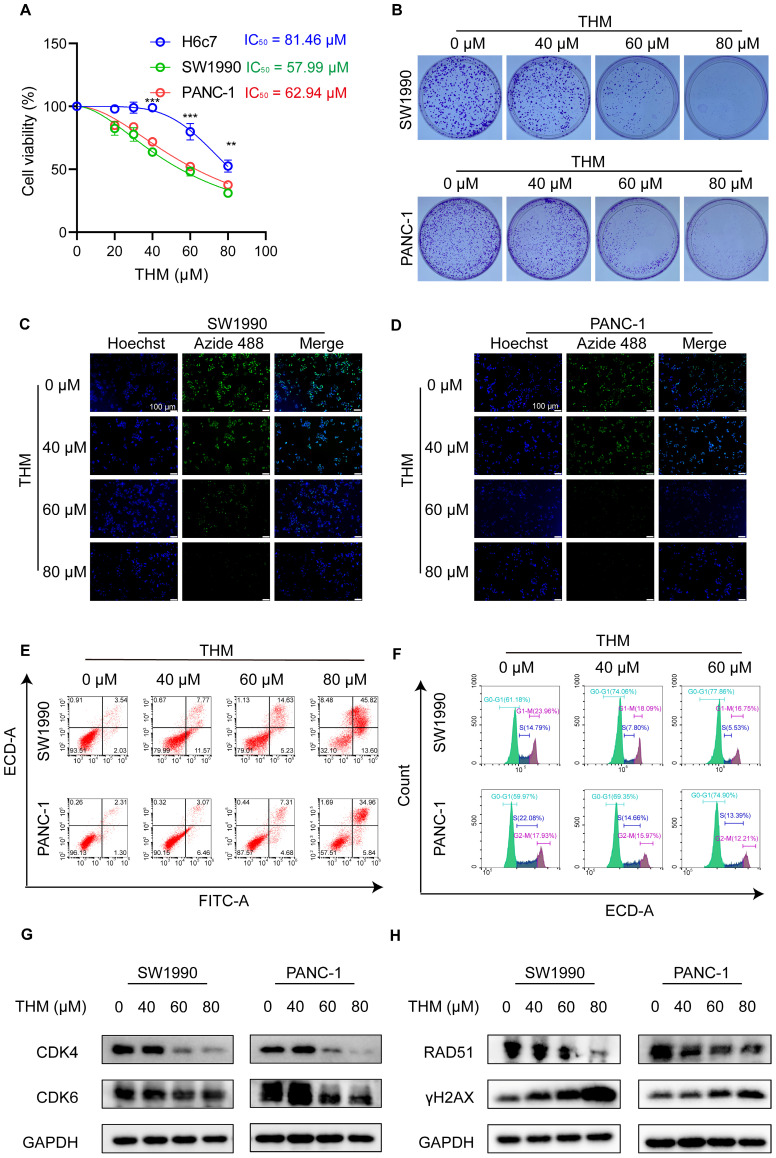
THM inhibited cell proliferation and induced cell death in pancreatic cancer cells. (**A**) SW1990, PANC-1 and H6c7 cells were treated with different concentrations of THM for 24 h, and cell viability was measured by CCK8 assay. (**B**) The cell proliferation effects of SW1990 and PANC-1 cells with THM treatment were assessed by colony-formation assay. (**C-D**) Fluorescence images of EdU-488 showed reduced green fluorescence after THM treatment relative to the control group. Scale bars:100 μm. (**E**) Representative results of annexin V/FITC/PI staining of SW1990 and PANC-1 cells after THM treatment for 24 h. (**F**) SW1990 and PANC-1 cells were treated with THM for 24 h and the cell cycle distribution was analyzed by flow cytometry. (**G**)Western blot analysis of the expression of the cell cycle-related proteins after THM treatment for 24 h. (**H**)Western blot analysis of the expression of the DNA damage-related proteins after THM treatment for 24 h.

To assess the effect of THM on cell viability in both cancer cells and normal cells, the normal human pancreatic ductal epithelial cell line H6c7 and six pancreatic cancer cell lines were treated with various concentrations of THM for 24 h. As expected, the cytotoxicity assay results using the cell counting kit-8 (CCK-8) revealed that, compared to normal pancreatic ductal epithelial cells, THM was selectively cytotoxic to pancreatic cancer cells in a dose-dependent manner (**Figure 1A** and **Figure S1A**). The evaluation of the effect of THM on cell proliferation using a clone formation assay showed that THM effectively suppressed the proliferation of pancreatic cancer cells (**Figure 1B** and **Figure S1B-C**). Also, the results of the 5-ethynyl-2-deoxy-uridine (EdU) proliferation assay also showed that THM treatment significantly reduced pancreatic cancer cell proliferation (**Figure 1C-D**).

In addition, flow cytometry analysis, using dual Annexin V-fluorescein isothiocyanate (FITC)/propidium iodide (PI) staining, showed that THM treatment caused significant cell death in pancreatic cancer cell (**Figure 1E** and **Figure S1D-E**). To further investigate whether THM suppresses cell growth and proliferation by inducing cell cycle arrest, we conducted flow cytometry analysis and observed that THM treatment resulted in cell cycle arrest at the G0-G1 phase (**Figure 1F** and **Figure S1F-G**). Furthermore, the expression levels of cell cycle-associated proteins, including cyclin-dependent kinase 4 (CDK4), and cyclin-dependent kinase 6 (CDK6), were significantly downregulated in a dose-dependent manner, leading to cell cycle arrest (**Figure 1G**). Considering the close relationship between DNA damage and the cell cycle 23, the expression levels of DNA damage repair-associated proteins RAD51, and the DNA double-strand break marker protein γH2AX were also analyzed. After THM treatment, results indicated that the levels of RAD51 were notably decreased, while the level of γH2AX was markedly increased (**Figure 1H**). In addition, the γH2AX immunofluorescence assay showed significantly enhanced rH2AX fluorescence in the nucleus of the cells after THM treatment, suggesting that THM could induce DNA damage in pancreatic cancer cells (**Figure S1H-I**). These findings suggested that THM reduced cell viability, suppressed cell proliferation and triggered cell death in pancreatic cancer cells.

### Autophagic cell death predominantly contribute to THM-triggered cell death in pancreatic cancer cells

**Figure 2:**
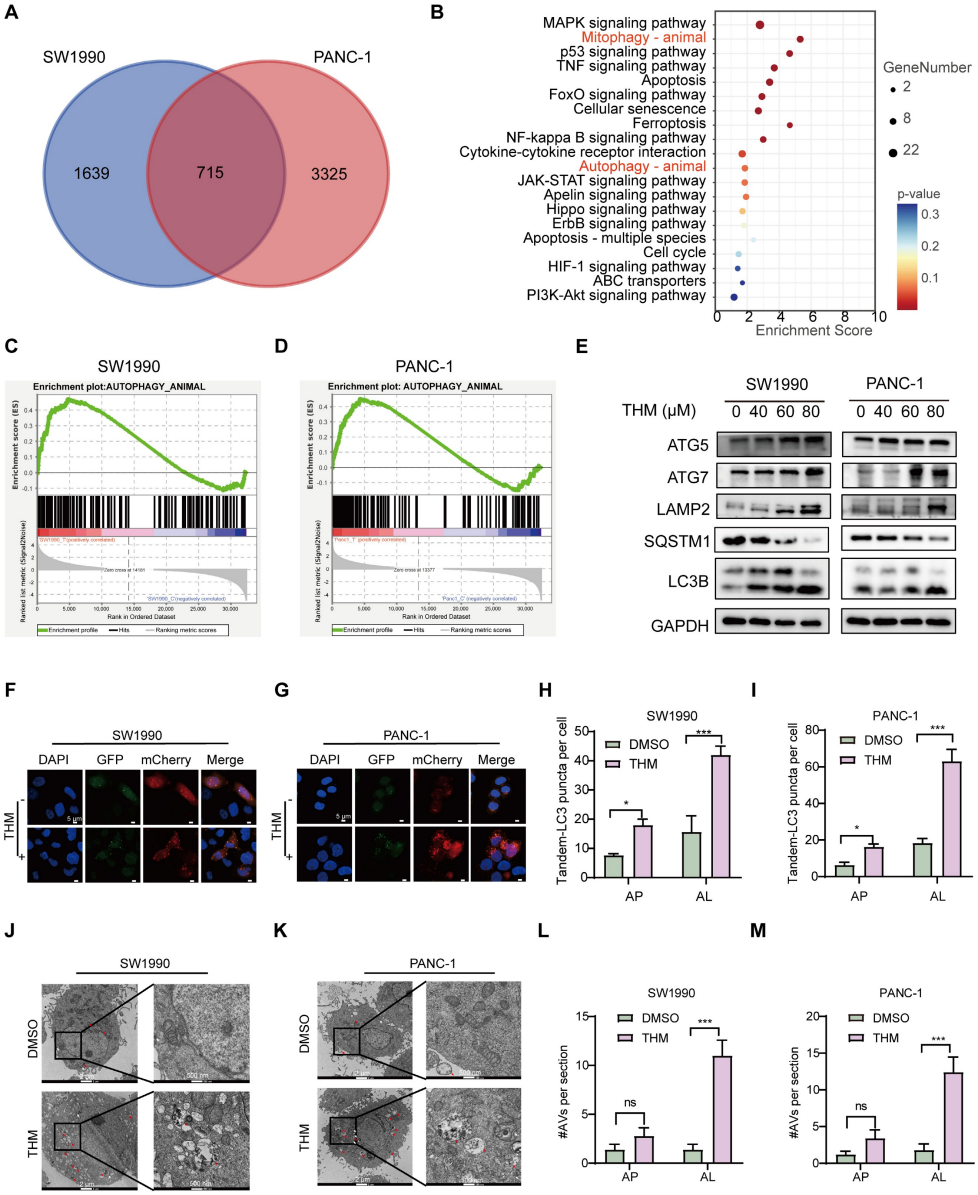
THM induced autophagic cell death in pancreatic cancer cells. (**A**) Venn diagram of THM versus control group in SW1990 and PANC-1 cells. (**B**) Pathways enrichment based on 715 differential genes expressed using Metascape in pancreatic cancer cell lines. (**C-D**) Transcriptome sequencing of SW1990 and PANC-1 cells with or without THM treatment and GSEA enrichment analysis showed high enrichment in the autophagy pathway. (**E**) Western blot analysis of the expression of the autophagy-related proteins following THM treatment for 24 h. (**F-G**) The effect of THM on the induction of autophagy in pancreatic cancer cells was analyzed by transfection of mCherry-GFP-LC3 labelled plasmids in SW1990 and PANC-1 cells by using laser confocal microscopy. Scale bars:5 μm. (**H-I**) Quantification of representative fluorescence micrographs, including AP (autophagosome) and AL (autolysosome). (**J-K**) Representative electron micrographs of DMSO or THM treatment SW1990 and PANC-1 cells. Red arrows indicate AL, and white arrows indicate AP. (**L-M**) Quantification of AVs (autophagic vesicles, including AP and AL).

We further investigated the specific signaling pathways mediating THM-induced cell death using transcriptome analysis. Visualization of transcriptomic data through Venn diagrams revealed that in SW1990 and PANC-1 cells, 715 differentially expressed genes (DEGs) were present in the THM group compared to their respective controls (**Figure 2A**). Subsequent Kyoto Encyclopedia of Genes and Genomes (KEGG) functional enrichment analysis of the 715 DEGs revealed that THM treatment significantly affected the autophagy pathway (**Figure 2B**). In addition, through gene set enrichment analysis (GSEA) of these DEGs, it was found that the autophagy pathway was enriched in cells treated with THM (**Figure 2C-D**). To determine the main form of cell death triggered by THM treatment, we used various inhibitors targeting distinct cell death pathways. It was found that the pan-caspase inhibitor Z-VAD-FMK, ferroptosis inhibitor deferoxamine (DFO), necroptosis inhibitor necrostatin-1 (Nec-1), or reactive oxygen species (ROS) scavenger N-acetyl cysteine (NAC) failed to significantly reverse the THM-induced cell death in pancreatic cancer cells (**Figure S2A-D**). Conversely, autophagy inhibitor chloroquine (CQ) and Bafilomycin A1 (Baf-A1) remarkably rescued THM-triggered cell death (**Figure S2E-F**). Additionally, to confirm whether THM induced autophagy in pancreatic cancer cells, we further assessed the expression levels of multiple autophagy-related proteins, including ATG5, ATG7, LAMP2, SQSTM1 and LC3B, in THM-treated cells. The results revealed that the expression levels of autophagy-related proteins gradually increased after THM treatment. The expression of the typical autophagy receptor SQSTM1 was significantly reduced after THM treatment, which reflected the patency of autophagic flow (**Figure 2E**). We also analyzed the formation of autophagic vesicles (including (autophagosome and autophagosomes) using an mCherry-GFP-LC3 expression system. Remarkably, there was a significant increase in the red puncta (autolysosomes) in THM-treated SW1990 and PANC-1 cells, which further confirmed the autophagy flux-promoting effect of THM (**Figure 2F-I**). Furthermore, transmission electron microscopy (TEM) imaging showed significant autolysosome formation in SW1990 and PANC-1 cells after THM treatment (**Figure 2J-M**). In addition, to further determine whether autophagy exerts a critical role in the THM-treated cells, we knocked down the expression of ATG5 or ATG7 (**Figure S3A-D**) and measured cell viability after exposing cells to different THM concentrations using the CCK-8 assay. The results showed that silencing ATG5 or ATG7 significantly boosted cell viability after THM treatment (**Figure S3E-H**). In addition, we performed overexpression experiments for ATG5 and ATG7. Western blot analysis confirmed the successful overexpression of ATG5 and ATG7 in the SW1990 and PANC-1 cells (**Figure S3I-L**). Subsequent evaluation of sensitivity to THM in these ATG5 or ATG7 overexpressing cells revealed that the overexpression of ATG5 or ATG7 significantly enhanced THM-induced autophagic cell death (**Figure S3M-P**). All these data suggested that THM primarily induced cell death in pancreatic cancer cells by autophagy.

### YTHDF2 was a direct cellular target of THM

**Figure 3:**
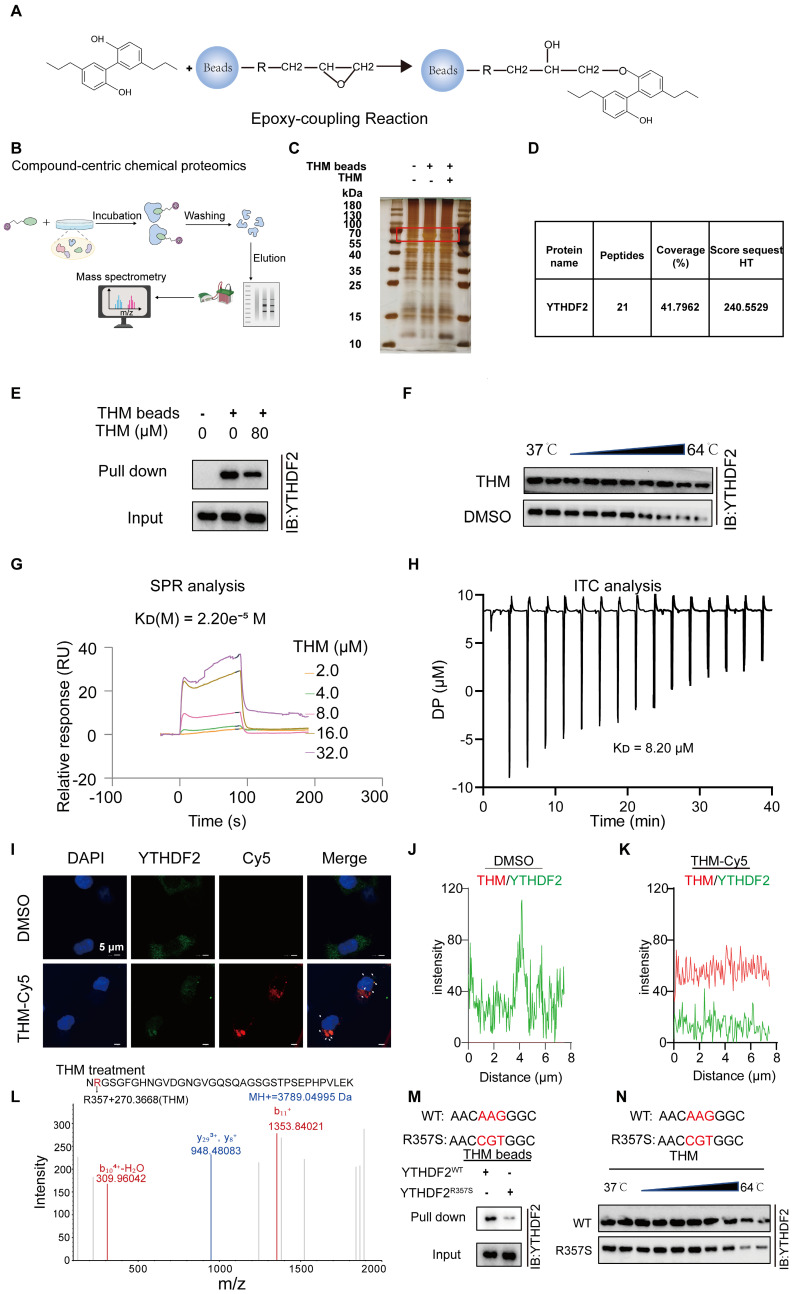
YTHDF2 was a direct cellular target of THM. (**A**) Schematic synthesis of THM-beads synthesized by incubation of epoxy-based coupled magnetic beads with THM for pull-down experiments. (**B**) Overall schematic diagram of the Compound-centric chemical proteomics method.** (C)** Identification of specific binding proteins by THM beads using silver staining.** (D)** Identification of specific binding proteins by THM beads using mass spectrometry. (**E**)THM conjugated beads were incubated with SW1990 cells lysates in the presence or absence of excess THM. (**F**) THM promoted the resistance of YTHDF2 to different temperature gradients by CETSA in SW1990 cells. (**G**) YTHDF2 exhibited a potent binding ability with THM determined by a SPR assay. (**H**) YTHDF2 exhibited a potent binding ability with THM determined by an ITC assay. (**I**) Co-localization of THM-Cy5 (red) and YTHDF2 (green) by immunofluorescence analysis (bar = 5 μm). White arrows indicate overlapped signals. (**J-K**) Intensity plots depicting fluorescent intensity (y-axis) versus distance (x-axis) illustrate the overlap between channels in DMSO or THM-Cy5-treated SW1990 cells. (**L**) Typsin-digest LC-MS/MS analysis indicated modification of YTHDF2 by THM at residue Arg357. Recombinant YTHDF2 protein was treated in THM at 24 °C for 2 h. (**M**) Schematic design of YTHDF2 mut (R357S) plasmids (upper), R357S blocked the interaction of THM with YTHDF2. Recombinant YTHDF2 protein and its mutants were incubated with THM-beads for pull-down experiments (bottom). (**N**) Schematic design of YTHDF2 R357S plasmids (upper), the CETSA was performed by using recombinant YTHDF2 WT/R357S proteins in the presence of THM. The stability of YTHDF2 proteins under 37-64 °C was measured by western blot (bottom).

To determine the potential target of THM, THM-conjugated epoxy-activated Sepharose beads (THM beads) were prepared and used to capture proteins that directly bind to THM, which are likely responsible for mediating its anticancer effect (**Figure 3A**). The results revealed a distinct THM-binding protein band that was confirmed to correspond to YTHDF2 by sodium dodecyl sulfate-polyacrylamide gel electrophoresis (SDS-PAGE) staining and mass spectrometry (MS) analyses (**Figure 3B-D**). A subsequent pull-down assay confirmed that the protein captured by THM beads from cell lysates was the YTHDF2 protein (**Figure 3E**). The evaluation of effect of THM on the stability of YTHDF2 by cellular thermal shift (CETSA) assays revealed that THM increased the stability of the YTHDF2 protein within a temperature range of (37-64 °C) (**Figure 3F**). Furthermore, a quantitative SPR analysis showed that THM exhibited a relatively strong binding affinity with YTHDF2, with a dissociation constant (KD) of 2.20e-5 M (**Figure 3G**). Also, isothermal titration calorimetry (ITC) analysis showed that THM directly interacted with YTHDF2 (**Figure 3H**). We further investigated the drug-target binding affinity of YTHDF2 in pancreatic cancer cells by immunofluorescence staining using Cy5-labeled THM (THM-Cy5). The results showed partial co-localization between THM-Cy5 (red) and YTHDF2 (green), appearing as yellow, which suggests a possible direct interaction between them in the cells (**Figure 3I-K**). In addition, we further investigated how THM binds to YTHDF2, by liquid chromatography-tandem/MS (LC-MS/MS) analysis and found that THM can bind to Arg-357 in YTHDF2 (**Figure 3L**). To further substantiate the interaction between THM and Arg-357 in YTHDF2, YTHDF2 residue mutants were constructed and characterized to provide a comprehensive functional characterization of this critical residue. In **Figure 3M**, a pull-down assay demonstrated that overexpressed YTHDF2 with Arg-357 mutated to arginine (R357S) was less efficiently pulled down compared to wild-type YTHDF2. Additionally, as shown in **Figure 3N**, the evaluation of the binding affinity between the YTHDF2 Arg-357 mutant protein and THM by CETSA demonstrated a notable reduction in the thermal stability of the YTHDF2 R357S protein when bound to THM. These findings collectively highlight the importance of Arg-357 in YTHDF2 for its interaction with THM and indicate that THM directly targets YTHDF2.

### Molecular dynamics (MD) simulations of YTHDF2-THM protein-ligand complexes

**Figure 4:**
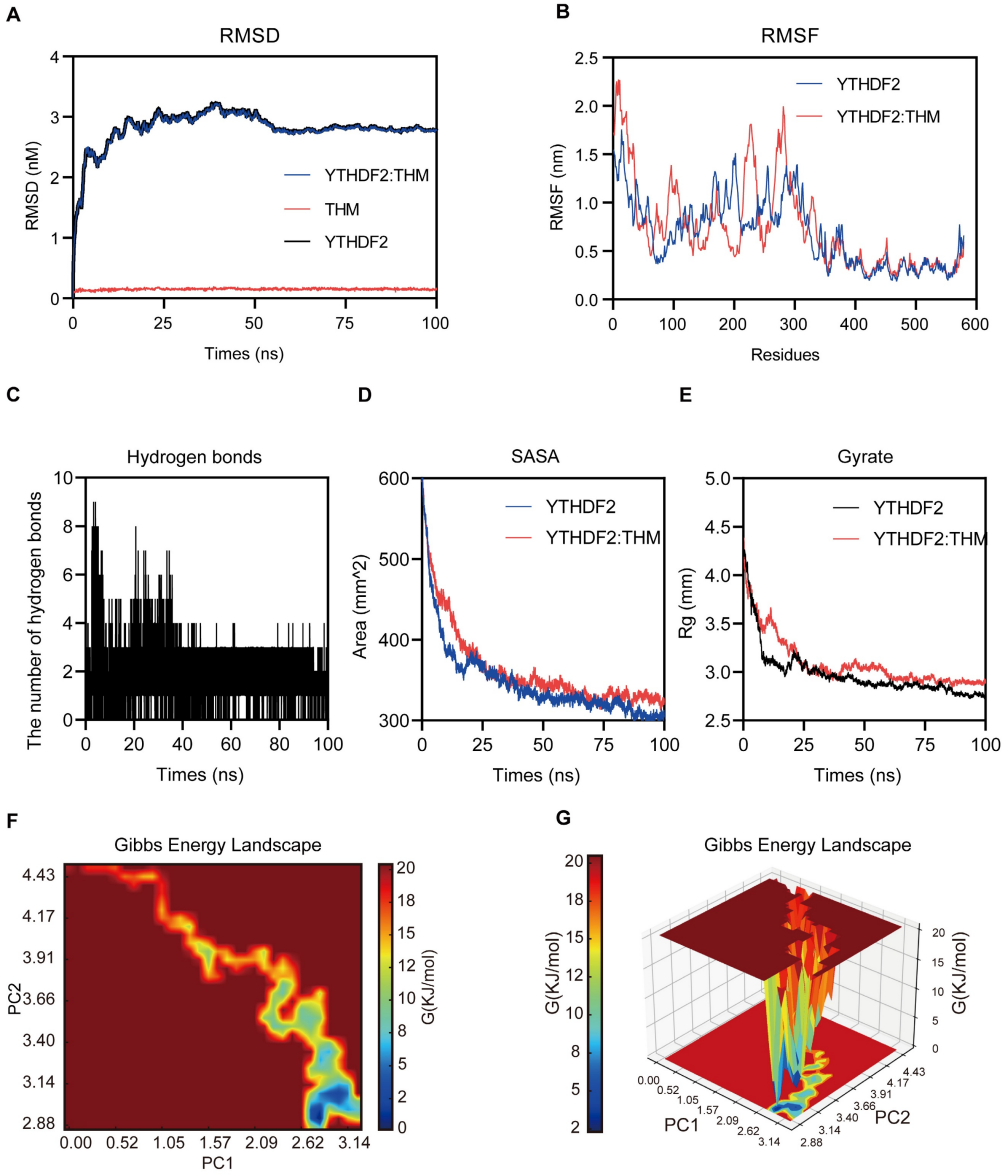
Molecular dynamics simulations of YTHDF2-THM protein-ligand complexes. (**A**) The binding mode of THM binding to YTHDF2 was simulated using molecular dynamics, and the figure shows the root-mean-square deviation (RMSD) values during 100 ns MD simulations. (**B**) The figure shows Root Mean Square Fluctuation (RMSF**)** values during MD simulation for residues 0-579. (**C**) Number of hydrogen bonds in ligand-protein complexes during 100 ns MD simulations. (**D**) SASA (solvent-accessible surface area) analysis during 100 ns MD simulations. (**E**) Mean square radius of gyration (Rg) analysis of proteins during 100 ns MD simulations. (**F**) 2D results of free energy landscaping (FEL) of proteins during 100 ns MD simulations. (**G**) 3D results of free energy landscaping (FEL) of proteins during 100 ns MD simulations.

The molecular dynamics (MD) simulations were performed to predict the binding modes of THM with YTHDF2. In these MD simulations, the calculation and plotting of the root mean square deviation (RMSD) between YTHDF2 and THM revealed that the value of the RMSD between YTHDF2 and THM was large during the first 10 ns of the MD simulation, and then gradually stabilized (**Figure 4A**). Overall, the small range of RMSD values indicated the relatively stable binding between YTHDF2 and THM. We calculated the root mean square fluctuation (RMSF) of YTHDF2 with and without THM to assess the protein dynamics. The results showed that the RMSF of YTHDF2 was lower in the bound regions and higher in the unbound regions, indicating that THM binding affects the protein stability (**Figure 4B**). Additionally, the MD simulation results also revealed that many hydrogen bonds (HBonds) formed between YTHDF2 and THM, which were dominated by HBonds between some key residues in YTHDF2 and some important groups in THM (**Figure 4C**). We also measured the surface area of the YTHDF2 protein by calculating its solvent-accessible surface area (SASA) value. The results indicated that the SASA value of YTHDF2 was larger before binding to THM and decreased after binding to THM, indicating that the binding to THM resulted in a decrease in the surface area of the YTHDF2 protein (**Figure 4D**). We also assessed the overall compactness of the YTHDF2 protein, based on its calculated gyrate value of YTHDF2. The results revealed that the gyrate value of YTHDF2 combined with THM was lower than that of the protein molecule alone, indicating that the combination with THM made the YTHDF2 molecule more compact (**Figure 4E**). The free energy landscape (FEL) results indicated the presence of two relatively stable conformations in the THM-YTHDF2 complex system (**Figure 4F-G**). Overall, all these findings indicated the complex formed by the binding of YTHDF2 and THM.

### THM increased the cytotoxicity of pancreatic cancer cells through targeting YTHDF2 protein

**Figure 5:**
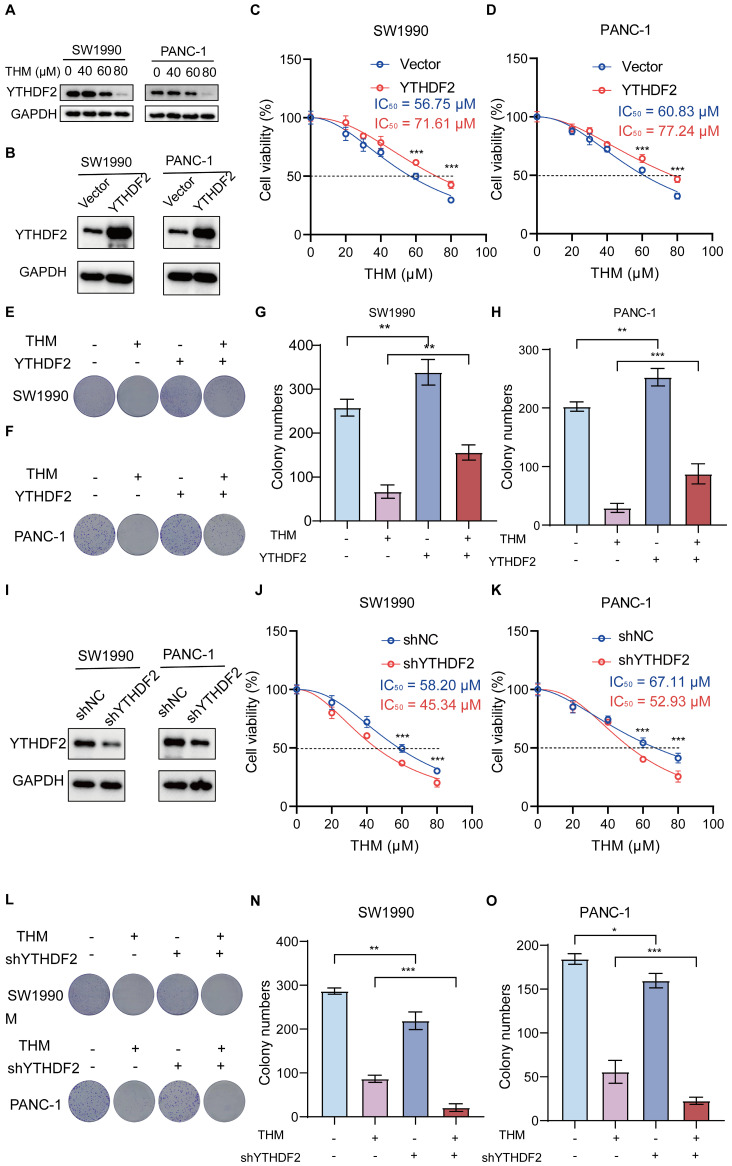
THM increased the cytotoxicity of pancreatic cancer cells through targeting YTHDF2 protein. **(A)** The expression of YTHDF2 by western blot after treatment of SW990 and PANC-1 with THM for 24 h.** (B)** YTHDF2 protein in stable over-expressed SW1990 and PANC-1 cells was analyzed by western blot. GAPDH was used as internal control. **(C-D)** CCK8 analysis was performed to investigate the cell viability of SW1990 and PANC-1 subjected to stable over-expression of YTHDF2 with the indicated doses of THM treatment for 24 h. ***p < 0.001 (n = 3). **(E-H)** The cell proliferation effects of SW990 and PANC-1 over-expressed YTHDF2 cells with THM treatment were assessed by colony-formation assay. Data are summarized as mean ± S.D., *p < 0.05, **p < 0.01, ***p < 0.001 (n = 3).** (I)** YTHDF2 protein in stable knocked-down SW1990 and PANC-1 cells was analyzed by western blot. GAPDH was used as internal control.** (J-K)** CCK8 analysis was performed to investigate the cell viability of SW1990 and PANC-1 subjected to stable knocked-down of YTHDF2 with the indicated doses of THM treatment for 24 h. ***p < 0.001 (n = 3). **(L-O)** The cell proliferation effects of SW990 and PANC-1 knocked-down YTHDF2 cells with THM treatment were assessed by colony-formation assay. Data are summarized as mean ± S.D., *p < 0.05, **p < 0.01, ***p < 0.001 (n = 3).

We also investigated whether the binding of THM to YTHDF2 affected the accumulation level of YTHDF2 protein in SW1990 and PANC-1 cells and observed that YTHDF2 protein levels notably decreased after THM treatment (**Figure 5A**).

In addition, to confirm that YTHDF2 is the dominant THM-target protein in the THM-induced cell death in pancreatic cancer cells, we generated stable cell lines overexpressing YTHDF2 by lentiviral infection (**Figure 5B**). We found that the IC_50_ values of THM were increased in YTHDF2-overexpressing SW1990 and PANC-1 cells, suggesting that overexpression of YTHDF2 reduced the sensitivity of cells to THM (**Figure 5C-D**). Additionally, the colony formation assay indicated that YTHDF2 overexpression notably diminished THM's inhibitory effects in SW1990 and PANC-1 cells (**Figure 5E-H**). Moreover, the stable knockdown of YTHDF2 in SW1990 and PANC-1 cells (**Figure 5I**) significantly reduced cell viability after THM treatment for 24 h (**Figure 5J-K**). Furthermore, colony formation assays revealed that reduced YTHDF2 expression significantly enhanced the THM's inhibitory effect on SW1990 and PANC-1 cells (**Figure 5L-O**). These results implied that YTHDF2 is a major target protein of THM in pancreatic cancer cells, and YTHDF2 overexpression promoted cell viability and proliferation of TMH-treated cells, whereas YTHDF2 knockdown inhibited their viability.

### THM suppressed YTHDF2 recognition of m^6^A mRNA targets

**Figure 6:**
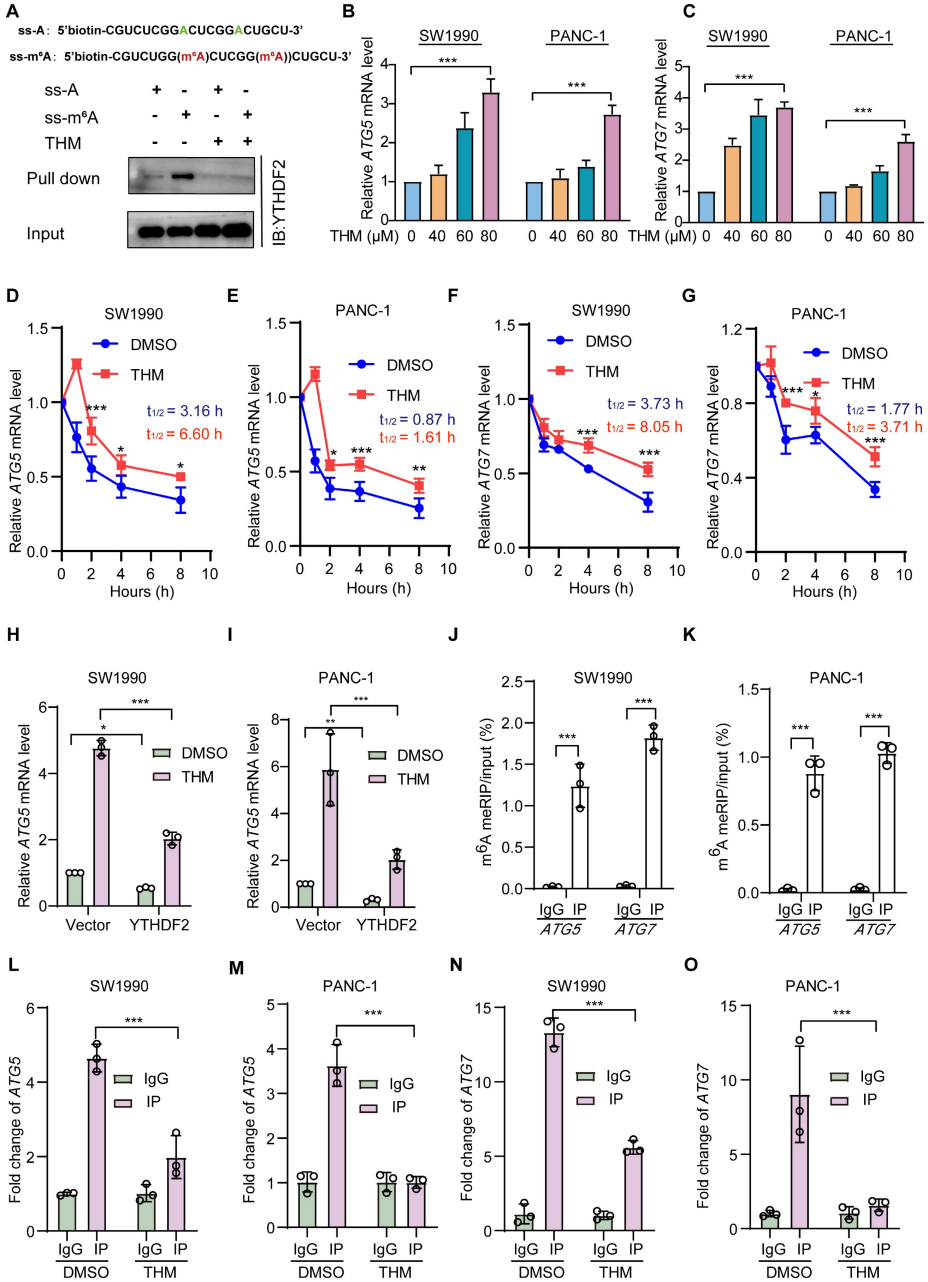
THM suppressed YTHDF2 recognition of m^6^A mRNA targets. (**A**) Schematic design of ss-m^6^A probe primers (upper), THM inhibited YTHDF2 interaction with the ss-m^6^A probe using the RNA pull-down assay (bottom). (**B-C**) Detection of *ATG5* and *ATG7* mRNA expressions in SW1990 and PANC-1 cells after THM treatment for 24 h. Data were expressed as mean ± S.D. **p < 0.01, ***p < 0.001 vs. 0 μM group (n = 3). (**D-G**) *ATG5* and *ATG7* mRNA stability in SW1990 and PANC-1 cells upon THM treatment was assessed using qPCR in conjunction with actinomycin D treatment, an inhibitor of transcription. (**H-I**) Analysis of *ATG5* mRNA expression in SW1990 and PANC-1 cells by RT-qPCR after overexpression of YTHDF2 with or without THM treatment. Data were expressed as mean ± S.D. *p < 0.05, **p < 0.01, ***p < 0.001 (n = 3). (**J-K**) The relative levels of m^6^A in *ATG5* and *ATG7* mRNA were measured by MeRIP-qPCR from SW1990 and PANC-1 cells. Data were expressed as mean ± S.D. ***p < 0.001 (n = 3). (**L-O**) RIP-qPCR analysis showed enrichment of YTHDF2 for *ATG5* and *ATG7* transcripts in SW1990 and PANC-1 cells after THM treatment for 24 h. Data were expressed as mean ± S.D. ***p < 0.001 (n = 3).

To assess whether THM-mediated YTHDF2 regulation had a regulatory effect on m^6^A targets, we performed RNA pull-down assays with methylated single-stranded RNA (ss-m^6^A) as bait and unmethylated single-stranded RNA (ss-A) as control. Notably, our findings indicated that YTHDF2 exhibited a marked preference for binding the ss-m^6^A probe over the ss-A probe, and this preference was reversed after treatment with THM, which might prevent the binding of YTHDF2 to mRNA (**Figure 6A**). A previous study found that YTHDF2 regulated autophagy by reducing the half-life of m^6^A-modified mRNAs to reduce ATG5 and ATG7 protein expression 21. Therefore, we assessed the mRNA expression levels of *ATG5* and *ATG7* after treatment with THM and found a notable upregulation of the expression these genes in SW1990 and PANC-1 cells (**Figure 6B-C**). Additionally, we also found that THM treatment significantly increased the half-life of *ATG5* and *ATG7* mRNAs (**Figure 6D-G**). Moreover, it was confirmed that THM had an inhibitory effect on the YTHDF2-mediated mRNA decay. Our results suggested that treatment with THM significantly increased the abundance of the *ATG5* and *ATG7* transcripts, but it was reduced in YTHDF2 overexpressing cells (**Figure 6H-I**, **Figure S4A-B**). Remarkably, the knockdown of *ATG5* and *ATG7* did not influence the expression levels of YTHDF2, which suggested that the THM-induced upregulation of *ATG5* and *ATG7* was indeed mediated by YTHDF2 (**Figure S4C-J**). To further determine whether m^6^A modifications are present in *ATG5* and *ATG7* mRNAs, we examined the level of m^6^A modifications in *ATG5* and *ATG7* mRNAs by methylated-RNA immunoprecipitation-quantitative polymerase chain reaction (MeRIP-qPCR) analysis. We first predicted potential m^6^A sites on ATG5 and ATG7 using the sequence-based RNA adenosine methylation site predictor (SRAMP) tool and designed primers for the highest scoring sites (**Figure S4L-L**). Subsequently, MeRIP-qPCR analysis showed that *ATG5* and *ATG7* mRNAs could be enriched using the m^6^A antibody (**Figure 6J-K**). In addition, RNA immunoprecipitation (RIP) analysis found that YTHDF2 strongly interacts with *ATG5* and *ATG7* mRNAs. Also, the enrichment of *ATG5* and *ATG7* transcripts for YTHDF2 was significantly reduced after THM treatment (**Figure 6L-O**). Together, these findings support the conclusion that THM reduces m^6^A recognition by YTHDF2 in *ATG5* and *ATG7* mRNAs. Furthermore, we demonstrated that knockdown of *YTHDF2* increases cell death, which can be reversed by the autophagy inhibitor CQ (**Figure S5A-B**), further linking the THM/YTHDF2 pathway to autophagic cell death. Our findings collectively suggested that THM modulated autophagy through the regulation of the m^6^A modification of *ATG5* and *ATG7* mRNAs by YTHDF2, thereby influencing their stability and expression. This mechanistic insight demonstrated the potential of targeting the m^6^A regulatory pathway to regulate autophagy and cell death in pancreatic cancer cells.

### THM inhibited pancreatic cancer tumor growth *in vivo*

**Figure 7:**
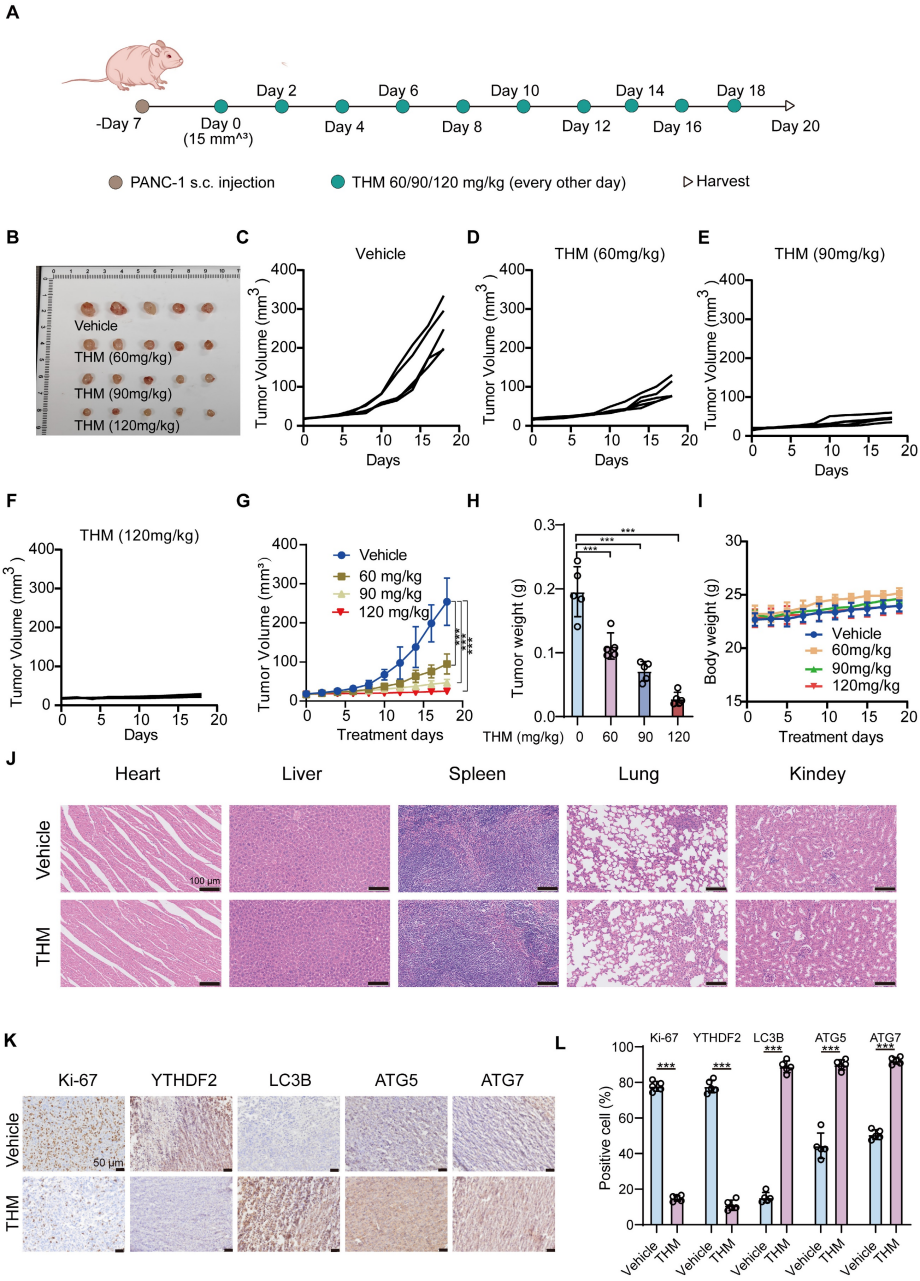
THM suppressed pancreatic cancer tumor growth *in vivo*. (**A**) Schematic representation of THM administration in mice with established PANC-1 tumors. THM (60,90 or 120 mg/kg) was administered intraperitoneally to BALB/c nude mice bearing PANC-1 xenografts every other day for 10 times. (**B**) Images of tumor samples in each group (n = 5). (**C-F**) Individual tumor growth kinetics in vehicle (C), 60 mg/kg THM (D), 90 mg/kg THM (E), 120 mg/kg THM group (F) (n = 5). (**G**) The dynamic change of tumor volume in subcutaneous models was shown. (**H**) The tumor weight of BALB/c nude mice was shown. (**I**) The dynamic change of body weight of BALB/c nude mice was shown. (**J**) Representative histological analysis of H&E-stained heart, liver, spleen, lung, and kidney specimens (scale bar = 100 μm). (**K**) Immunohistochemical (IHC) analysis of Ki-67, YTHDF2, LC3B, ATG5 and ATG7 expression (scale bar = 50 μm). (**L**) Quantification of IHC staining is shown (n = 5). Black arrows indicate positive staining cells. Data are shown as mean ± S.D. ***p < 0.001 vs. vehicle group.

To assess whether THM inhibited tumor growth *in vivo*, we examined its effects in a PANC-1 cell xenograft model in BALB/c nude mice. A schematic of the experimental protocol is shown in **Figure 7A.** After injecting THM intraperitoneally, at different doses (60, 90 or 120 mg/kg), every other day for 10 consecutive doses, a significant inhibition was observed in the THM-treated groups (**Figure 6B-H**). Additionally, no statistically significant body weight disparity was noted between the vehicle and THM-treated groups (**Figure 7I**), and there were no significant toxic side effects (**Figure 7J** and **Figure S6A-E**). Further evaluation of the effects of THM on cell proliferation, autophagy, and the m^6^A reader protein YTHDF2 by immunohistochemistry (IHC) analysis revealed that THM decreased the expressions of Ki-67 and YTHDF2, and increased the expressions of ATG5, ATG7, and LC3B (**Figure 7K-L**), suggesting a possible mechanism by which THM inhibits the growth of pancreatic cancer.

**Figure 8:**
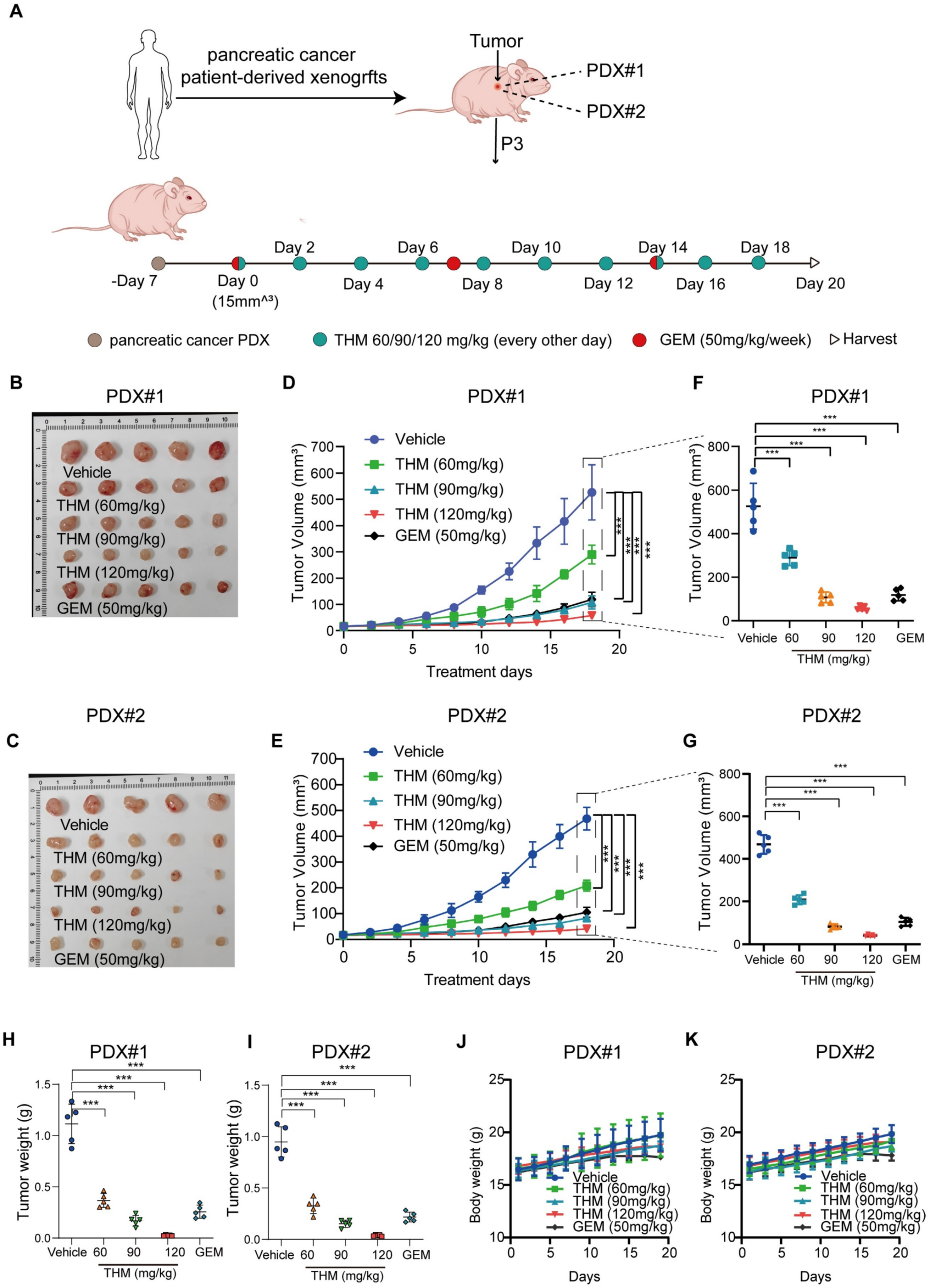
*In vivo* antitumor efficacy of THM and GEM in a preclinical model of pancreatic cancer. (**A**) Schematic diagram of generation and treatment of pancreatic cancer in PDX. (**B-C**) Images of tumor samples in PDXs across treatment groups were taken at the experimental endpoint (n = 5). (**D-E**) The dynamic change of tumor volume in PDXs models' mice was shown. Data are expressed as mean ± S.D. ***p < 0.001. (**F-G**) Tumor volumes at the experimental endpoint for all groups, data are expressed as mean ± S.D. ***p < 0.001. (**H-I**) The tumor weights in PDXs models' mice were measured across treatment groups were taken at the experimental endpoint. Data were expressed as mean ± S.D. ***p < 0.001 (n = 3). (**J-K**) The dynamic change of body weight in PDXs models' mice were shown.

In summary, these results suggested that THM inhibited pancreatic cancer tumor growth and induced autophagy *in vivo*. Furthermore, the evaluation of the therapeutic effect of THM and GEM in Patient-Derived Xenograft (PDX) tumor models, using the experimental protocol schematically shown in **Figure 8A**, revealed that compared to saline-treated PDX tumor-bearing mice, THM showed a dose-dependent antitumor activity. Moreover, THM treatment at moderate concentration (90 mg/kg) showed a similar antitumor activity as GEM treatment (**Figure 8B-I**). Additionally, no statistically significant body weight disparity was noted between the vehicle and THM-treated groups (**Figure 8J-K**), and there were no significant toxic side effects (**Figure S7A-J**).

### THM increased the sensitivity of pancreatic cancer tumors to anti-PD-1 treatment

**Figure 9:**
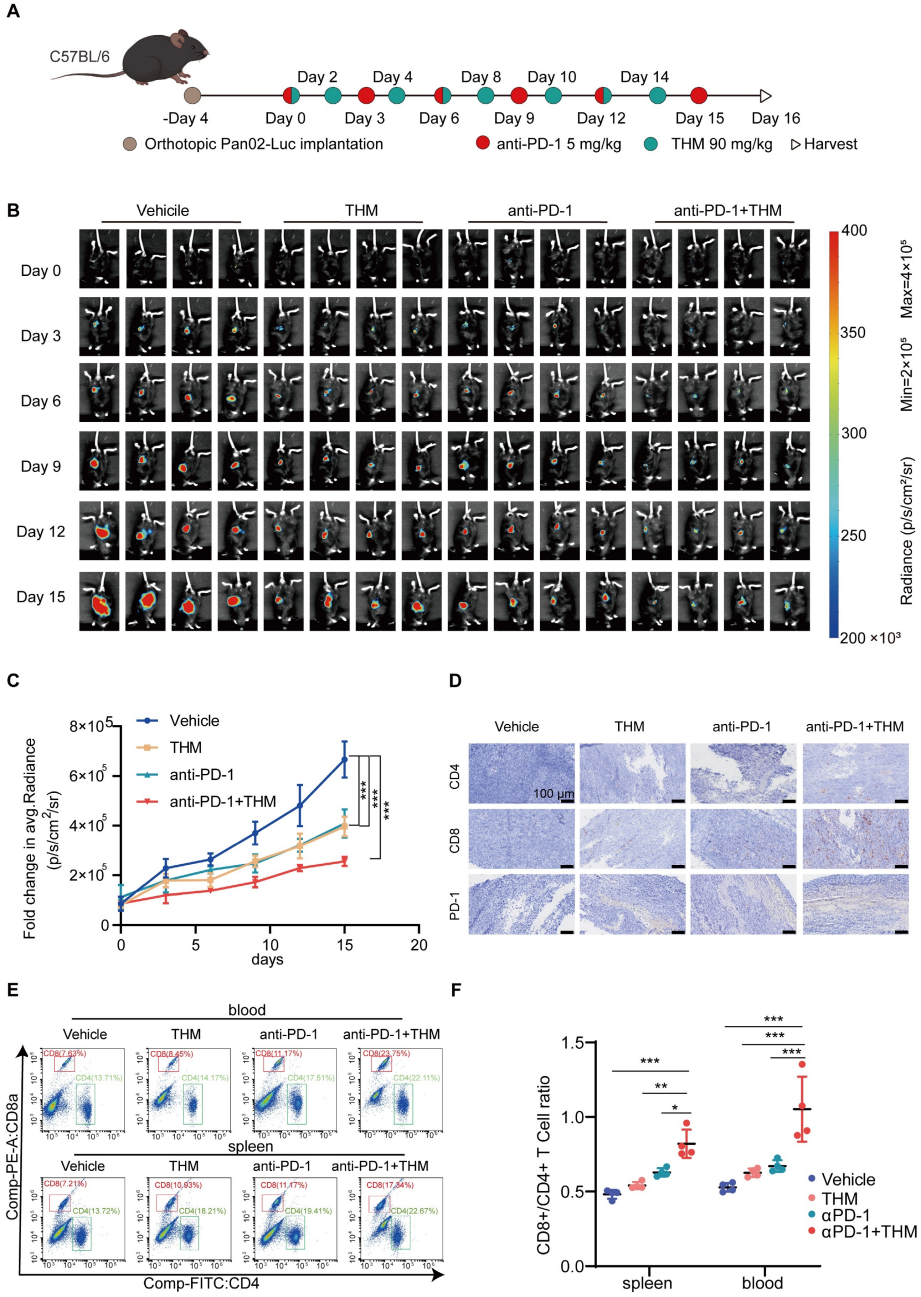
THM improved the sensitivity of pancreatic cancer tumors to anti-PD-1 treatment. (**A**) The Schematic diagram involved Orthotopic Pan02-Luc tumor inoculation and treatment scheduling in C57BL/6J mice. Four days after tumor inoculation, mice were treated daily with THM (90 mg/kg) every other day and αPD-1 (5 mg/kg) every three days. (**B**) Orthotopic Pan02-Luc pancreatic cancer tumor growth was monitored through evaluating the average radiance within the tumor sites by bioluminescence imaging on days 0, 3, 6, 9, 12, 15 and 18. (**C**) Fold changes in average radiance of mice at different timepoints treated with THM, anti-PD-1 or both. Data are represented as mean ± S.D., ***p < 0.001 (n = 4). (**D**) Immunohistochemical staining of CD4, CD8a and PD-1 of orthotopic Pan02-Luc pancreatic cancer tumor section. (**E-F**) Representative flow cytometry analysis (E) and quantitative (F) flow cytometry results for CD8a/CD4 expression in blood and spleen of orthotopic Pan02-Luc pancreatic cancer tumor and treated with vehicle, THM, αPD-1, or the combination. Data are summarized as mean ± S.D., *p < 0.05, **p < 0.01, ***p < 0.001 (n = 4).

Autophagic cell death, a form of non-apoptotic regulated cell death (RCD) also regulates the function of immune cells (such as T cells and dendritic cells) and activates immune cell death (ICD) to augment immunotherapeutic responses to PD-1/PD-L1 inhibition 22-24. Recent studies have demonstrated that the autophagy pathway is closely associated with the processing of MHC-presented antigens, and that induction of autophagy generates CD8+ T cells with greater tumor clearance capacity *in vivo* 25*.* Thus, to further assess whether THM enhances the therapeutic effect of αPD-1 on tumors, we established an orthotopic pancreatic cancer model using stably luciferase-expressing Pan02 (Pan02-Luc) cells injected into C57BL/6J mice. C57BL/6J mice bearing palpable orthotopic tumors were administered vehicle, THM, αPD-1 or a combination of THM and αPD-1. Tumor growth was monitored by bioluminescence imaging. After four days, mice were randomized into different groups to receive THM every other day or αPD-1 every three days or THM combined with αPD-1, respectively (**Figure 9A**). THM and αPD-1 were injected intraperitoneally and bioluminescence imaging was performed on days 0, 3, 6, 9, 12, and 15 (**Figure 9B**). The results revealed that the THM + αPD-1 combination group exhibited significant inhibitory effects when compared with the vehicle group, THM and αPD-1 individual groups (**Figure 9C**). In addition, IHC analysis showed significantly higher expression of CD4, CD8a and PD-1 in orthotopic pancreatic cancer sections in the THM + αPD-1 group compared with the vehicle group, THM and αPD-1 groups (**Figure 9D, Figure S8A-C**). Flow cytometry analysis indicated a marked increase in the proportion of CD4+ and CD8+ T cells in spleen and blood was significantly higher in mice receiving THM monotherapy or in combination with αPD-1 (**Figure 9E-F**). Therefore, combining THM with αPD-1 has the potential to remodel tumor immune microenvironment, facilitating T cell function and infiltration, thereby activating immune cells with antitumor properties. To evaluate the potential correlation between the THM/YTHDF2 signaling pathway and PD-L1 expression, PD-L1 protein expression levels in SW1990 and PANC-1 cells treated with varying concentrations of THM were assessed by western blot analysis. The results revealed that PD-L1 protein expression decreased with increasing THM concentration (**Figure S9A**). Furthermore, YTHDF2 knockdown in SW1990 and PANC-1 cells led to an even more pronounced reduction in PD-L1 expression, especially in THM-treated cells (**Figure S9B**). These findings suggest that THM can downregulate PD-L1 expression in a YTHDF2-dependent manner. In pancreatic cancer tissue samples, PD-1 expressions levels were examined across different treatment groups (vehicle, THM alone, αPD-1 alone, and combined THM and αPD-1) by IHC. The combination of THM and αPD-1 led to the most significant increase in PD-1 expression compared to other treatment groups (**Figure 9D**). This result implies that the THM/YTHDF2 signaling pathway may influence the tumor immune microenvironment and enhance the efficacy of PD-1 therapy. Therefore, the THM/YTHDF2 pathway not only regulates autophagy but also affects the tumor immune microenvironment by altering PD-L1 expression, thereby potentially improving the efficacy of immunotherapies.

## Discussion

Currently, more than 200 compounds, including magnolol, honokiol, 4-methoxyhonokiol, and THM, have been extracted from *Magnolia officinalis*. Previous studies have shown magnolol and honokiol possess anti-inflammatory and anticancer activities 26, 27 , while THM acts through cannabinoid receptors, primarily as an agonist of peripheral CB2 receptors, which might influence the hypoalgesia associated with inflammation 12. However, there have been few studies on THM, and no studies about its anticancer potential have been reported to date. Given the extensive application of natural products from *Magnolia officinalis* in cancer research, we investigated the effect of THM both *in vivo* in mice and *in vitro* in pancreatic cancer cells. Our findings indicated that THM effectively suppressed cell proliferation and triggered cell death.

Typically, natural small molecule compounds induced cancer cell death through two parallel pathways (autophagy and apoptosis). Based on our understanding of autophagy, it plays an important role in the anti-cancer mechanism of natural small molecule compounds 28, 29. Our transcriptomics results showed that THM significantly induced autophagy signaling in pancreatic cancer cells. In addition, western blot analysis results also showed that LC3B, ATG5 and ATG7, the key proteins associated with autophagy, were significantly increased. Notably, LAMP2, a crucial protein in the molecular chaperone-mediated autophagy (CMA) pathway 30, was also significantly increased, suggesting that the effects of THM may be mediated through the CMA pathway. Our study also showed that THM, isolated from *Magnolia officinalis*, exerted its antitumor effects on pancreatic cancer cells by inducing autophagic cell death.

Elucidating targets of drugs from natural products will lead to the development of effective strategies for the treatment of cancer. For example, the identification of microtubule as the anticancer target of paclitaxel initiated a new era of application of microtubule inhibitors in cancer treatment 31. As a result of the rapid development of chemical proteomics, the protein targets for specific natural biologically active compounds are gradually being explored 32. We followed up on a previous study using ligand coupled epoxy-activated magnetic beads to capture a target protein from cell lysates, a compound-centric chemical proteomics method 33, 34. Using a pull-down assay, our study identified the m^6^A-reader YTHDF2 protein as the first cellular target of THM. Subsequently, through the use of CETSA, SPR analysis, ITC analysis, immunofluorescence staining, LC-MS/MS analysis and MD simulations, we further confirmed the binding of THM to the YTHDF2 protein.

Recently, the involvement of the m^6^A methylation modification in tumorigenesis and therapy of malignant tumors has received extensive attention. YTHDF2, as a major "reader" protein of m^6^A, has been demonstrated to markedly influence m^6^A methylation modification and its abnormal expression or function is closely associated with the occurrence and metastasis of various tumors 14. However, there are few reports of small molecule compounds targeting YTHDF2 for cancer therapy. Our study showed that THM decreases YTHDF2 protein levels in pancreatic cancer cells. Overexpression of YTHDF2 reduced THM sensitivity by increasing IC_50_ values and restoring colony formation, whereas YTHDF2 knockdown enhanced THM-induced growth inhibition. Notably, dysfunction of YTHDF2 led to impaired mRNA degradation of the autophagy-related genes *ATG5* and *ATG7*. We hypothesized that THM directly blocks methylation recognition on the mRNAs of *ATG5* and *ATG7* through binding inhibition of YTHDF2. Furthermore, MeRIP-qPCR analysis showed significant enrichment of *ATG5* and *ATG7* mRNAs with the m^6^A antibody, confirming the presence of m^6^A modifications on these transcripts. These findings establish YTHDF2 as a key target of THM in pancreatic cancer cells, with potential implications for developing YTHDF2-targeted therapies. Noteworthy, the THM treatment-induced dysfunction of YTHDF2 was verified by RNA stability measurement and RIP analysis. Additional studies found that the anticancer efficacy of a medium dose of THM was as good as GEM, while a high dose of THM was superior to GEM in its anticancer effects *in vivo*. Furthermore, our results suggest that THM induces autophagic cell death by targeting YTHDF2. YTHDF2 has been reported to mediate the immune-supervised escape by regulating CD8+ T cells 35. Additionally, autophagy is also closely related to the processing of MHC-presented antigens, and induction of autophagy generates CD8+ T cells with tumor-scavenging capacity 25, 36. Inspired by these findings, we examined impact of THM on antitumor immune responses and found that THM significantly promoted the infiltration of CD4+ and CD8+ T cells and enhanced their immune activation. However, the study has some limitations. While our results demonstrate that THM can enhance T cell infiltration and activation, the precise mechanisms by which THM modulates the broader immune microenvironment, including its effects on macrophages or other immune cells, remain unclear. Future studies should explore the potential of THM to regulate immunosuppressive elements like Tregs, myeloid-derived suppressor cells (MDSCs), and M2-like macrophages, which are critical in shaping the pancreatic tumor microenvironment.

Collectively, our findings revealed the potential mechanisms by which THM inhibited pancreatic cancer proliferation and induced autophagic cell death. More importantly, we found that THM specifically binds to YTHDF2 as a direct cellular target to block the proliferation of pancreatic cancer cells. Moreover, the inhibition of YTHDF2 prevented the reading of m^6^A methylation modifications on the *ATG5* and *ATG7* mRNAs, thereby blocking the decay of these mRNAs, and ultimately leading to autophagic cell death.

In conclusion, this study revealed that the natural small molecule compound THM showed significant antitumor activity by inducing autophagic cell death both *in vitro* and *in vivo*. Furthermore, THM was used as a chemical molecular probe to explore new drug targets in pancreatic cancer. Mechanistically, we found that THM mediated the post-transcriptional regulation of autophagy activation by targeting YTHDF2, thereby preventing the reading of the m^6^A modifications of *ATG5* and *ATG7* mRNAs, ultimately resulting in autophagic death in pancreatic cancer cells. Overall, our results suggested that THM not only has significant anticancer activity but also enhances the effect of αPD-1 immunotherapy. This study provides a potential future direction for the development of new drugs for the treatment of pancreatic cancer.

## Materials and Methods

### Chemicals and Reagents

THM (Cat. #: C18H22O2, 98% purity) was procured from Shanghai Yuanye Biotechnology Co., Ltd. FBS was purchased from Vistech (Sydney, Australia). DMEM was purchased from Basal Media Technologies Co., Ltd. (Shanghai, China). The fluorescent dye DAPI was purchased from Solarbio (Beijing, China). The EdU Cell Proliferation Kit, Hoechst 33342 staining kit, RIPA buffer, phenylmethylsulfonyl fluoride (PMSF), Protease inhibitor cocktail, and NP-40 lysis buffer were bought from Beyotime (Shanghai, China). DFO was purchased from Selleck Chemicals (Houston, USA). Z-VAD-FMK, Ferrostatin-1, Nec-1, NAC, CQ and Actinomycin D were purchased from MCE. Lipofectamine 2000 was bought from Thermo Fisher Scientific. The ss-A and ss-m^6^A probes were designed by GenScript (Nanjing, China).

### Cell culture

SW1990, PANC-1, Capan-1, Capan-2, MIA-PACA-2, CFAPC-1 and H6c7 cells were acquired from the Cell Bank/Stem Cell Bank, Chinese Academy of Sciences (Beijing, China). These cells were regularly cultured in high-glucose DMEM or RPMI-1640 medium supplemented with 10% FBS and antibiotics (1% streptomycin and penicillin, both at 100 mg/mL) in a humidified incubator at 37 °C with 5% CO₂ to maintain optimal growth conditions.

### CCK-8 assay

For cell viability, SW1990/PANC-1 cells were enumerated, plated into 96-well plates (5,000 cells/well), and incubated overnight at 37 °C for adhesion. Fresh medium with gradient THM concentrations was added, and cells were cultured at 37 °C for 24 h. After incubation, a Thermo Fisher microplate reader measured OD at 450 nm. IC_50_ was calculated, and cell viability was quantified via GraphPad Prism 8.0 based on OD_450_ values.

### Colony-formation assay

For the colony-formation assay, SW1990 or PANC-1 cells were enumerated and seeded into 6-cm dishes at a seeding density of 3,000 cells/dish, and incubated overnight at 37 °C. Following the administration of THM at varying concentrations, cells were cultured for 10-15 days (medium refreshed every 3 days). Once visible colonies formed, cells were fixed and subjected to crystal violet staining for 2 h, and stained colonies were imaged.

### EdU staining

Cell proliferation was assessed *via* the EdU Kit per protocols protocols. Specifically, cells seeded in 6-well plates (5×10⁵ cells/well) were allowed to adhere, then treated with THM (varying concentrations) for 24 h. Next, cells were incubated with 10 μM EdU for 2 h, fixed in 4% paraformaldehyde for 15 min, permeabilized with 0.3% Triton X-100 for 15 min, and subjected to Click Additive Solution in the dark for 30 min. Subsequent Hoechst 33342 staining (10 min) was followed by PBS rinsing, slide mounting with a fluorescence quencher, and imaging via an upright fluorescence microscope.

### Cell cycle analysis

After exposing SW1990 or PANC-1 cells to different concentrations of THM for 24 h, the cells were collected and stained in a PI solution for 30 min. Then, the samples were subjected to flow cytometric analysis using a Beckman cytometer (Beckman, USA).

### Cell death analysis

For cell death analysis, SW1990 or PANC-1 cells were exposed to varying concentrations of THM for 24 h, then underwent staining with the Annexin V-FITC-PI Apoptosis Detection Kit (Yeasen, China). Subsequent cell death assessment was conducted *via* flow cytometry following the manufacturer's protocols.

### Western blot analysis, antibodies and plasmids

Western blot analysis was performed following established laboratory procedures, using primary antibodies against CDK4 (Cat. #: 12790; Cell Signaling Technology, CST), CDK6 (Cat. #: 3136; CST), RAD51 (Cat. #: 4937; CST), Phospho-Histone H2A.X (Ser139; Cat.: 80312; CST), YTHDF2 (Cat. #: 71283; CST), ATG5 (Cat. #: A19677; ABclonal Biotechnology Co., Ltd., Wuhan, China), ATG7 (Cat. #: A21895; ABclonal Biotechnology Co., Ltd.), LAMP2 (Cat. #: 59067; CST), SQSTM1/p62 (Cat. #: 23214; CST), LC3B (Cat. #: 3868; CST), PD-L1 (ABclonal Biotechnology Co., Ltd., A11273), GAPDH (Cat. #: 51740; 1:1,000; CST). Negative control (vector), YTHDF2-overexpressing, YTHDF2 knockdown and YTHDF2 mut (R357S) plasmids were designed by Tsingke (Beijing, China).

### Transmission electron microscopy (TEM)

For TEM analysis, SW1990 and PANC-1 cells were first subjected to 24 h of incubation at 37 °C with either DMSO (vehicle control) or THM. To preserve ultrastructural details, cells were immobilized in 2.5% glutaraldehyde at 4 °C, followed by post-fixation and contrast enhancement using 1% OsO_4_ for 1-2 h to enhance the contrast of cellular structures like the cell membrane. Subsequently, sample processing involved gradient dehydration (ethanol/acetone series), embedding in epoxy resin, and ultramicrotomy to generate 50 nm-thick sections, uranyl acetate staining (3 min), and visualization *via* a Tecnai Spirit 120 kV TEM.

### Cellular thermal shift assay (CETSA)

For CETSA, SW1990 cells incubated with DMSO or 80 μM THM at room temperature for 2 h were gradient-heated (37-64 °C, 3 min/temperature) *via* a PCR instrument, then subjected to five liquid nitrogen freeze-thaw cycles. After centrifugation, supernatant was collected, mixed with SDS loading buffer for subsequent western blot.

### Surface plasmon resonance (SPR) analysis

SPR analysis was conducted on a Cytiva Biacore™ 1k SPR system (Cytiva, Marlborough MA, USA) equipped with a Cytiva Series CM5 Sensor Chip at 25 °C. The recombinant protein of YTHDF2 (NCBI: 51441; designed by General Biology (Anhui, China) was covalently fixed on the surface of the chip through its amine groups in a firm manner. Different concentrations of THM (2-fold dilution from 64 to 4 μM) were run as mobile phase through the chip surface in 10 mM phosphate buffer containing 5% DMSO. Data were analyzed using Cytiva Biacore 1k evaluation software.

### Isothermal titration calorimetry (ITC) analysis

Binding affinity and thermodynamic characteristics of YTHDF2 protein and THM interactions were assessed using a MicroCal iTC200 isothermal titration calorimeter. The protein was titrated against 50 μM THM at 25 °C, and then the data were analyzed.

### Identification of THM-binding site on YTHDF2

Recombinant YTHDF2 (5 μM) was combined with THM (50 μM) and incubated at room temperature for 2 h, followed by trypsin digestion for 16 h. After vacuum drying, the tryptic peptides were processed *via* an EASY-LC™ system (Thermo Fisher) and analyzed by nanoflow LC-linear trap quadrupole MS. Elution (2-40% B over 70 min, 95% B for 20 min; Solvent A/B: 0.1% formic acid in water/ACN) was directly injected into a linear trap quadrupole MS at 300 μL/min. MS/MS targeted the top 15 precursor ions (m/z 350-2000) in a linear ion trap with 35% CID. Data were analyzed *via* SEQUEST in Proteome Discoverer (Thermo Fisher).

### Immunofluorescence (IF) assay

THM-Cy5 was synthesized by Qiyue Biological Technology (Xi'an, China). SW1990 cells were cultured in confocal dishes, incubated with 80 μM THM-Cy5 for 2 h, fixed in 4% paraformaldehyde, washed with PBS, permeabilized with 0.1% Triton X-100, and blocked with 5% BSA. After overnight incubation with YTHDF2 primary Ab (1:1000) at 4 °C, cells were incubated with Alexa Fluor 488-conjugated secondary antibody (1:200) for 1 h, and stained with DAPI for 15 min. Imaging was performed *via* an Olympus IX83-FV3000RS confocal microscope.

### MD simulation

In this study, MD simulations were performed using Gromacs 2022.3. Small molecules preparation was prepared using AmberTools22, in which the GAFF force field was deployed. Restrained Electrostatic Potential (RESP) charges were obtained through hydrogen addition and electrostatic potential calculations using Gaussian16W (hydrogen addition, electrostatic potential) and integrated into the MD system topology. Simulations ran at 300 K, 1 Bar (Amber99sb-ildn force field), with water molecules were modeled using the Tip3p model, and Na⁺ ions were introduced to neutralize the net charge of the system. The system underwent 100,000-step NVT (0.1 ps coupling, 100 ps) and 100,000-step NPT equilibration, followed by 50 million production steps (2 fs/time step, 100 ns total).

### RNA extraction and quantitative RT-PCR

Total RNA isolated *via* TRIzol was reversed-transcribed to cDNA using a qPCR synthesis kit. RT-qPCR ran in triplicate, normalized to GAPDH. Primer sequences for qPCR are listed: *YTHDF2*: 5ʹ-3ʹ (sense) AGCCCCACTTCCTACCAGATG, 5ʹ-3ʹ (antisense) TGAGAACTGTTATTTCCCCATGC; *ATG5*: 5ʹ-3ʹ (sense) AAAGATGTGCTTCGAGATGTGT, 5ʹ-3ʹ (antisense) CACTTTGTCAGTTACCAACGTCA; *ATG7*: 5ʹ-3ʹ (sense) CTGCCAGCTCGCTTAACATTG, 5ʹ-3ʹ (antisense) CTTGTTGAGGAGTACAGGGTTTT; *GAPDH*: 5ʹ-3ʹ (sense) AGCCACATCGCTCAGACAC, 5ʹ-3ʹ (antisense) GCCCAATACGACCAAATCC.

### RNA stability assay

After exposing SW1990 or PANC-1 cells to actinomycin D (5 μg/mL) with or without THM for the specified time periods, the cells were washed with PBS and collected. The lifespan of mRNA half-life at 50% mRNA decay (t_1/2_) was calculated using the ln 2/(-slope).

### MeRIP-qPCR

The m^6^A sites of *ATG5* and* ATG7* were deployed using the SRAMP database. For MeRIP-qPCR, the EpiQuik CUT&RUN m^6^A RNA Enrichment Kit (Epigentek) was used following the manufacturer's instructions. Total RNA was isolated, fragmented to approximately 100-200 nt fragments, and incubated overnight with a mixture of m^6^A antibody and protein G magnetic beads with continuous rotation. After removing nonspecific RNA by washing, the m^6^A-bound RNA was purified and used to synthesize cDNA for qPCR analysis. Relative m^6^A enrichment was determined by normalizing the input RNA. Primer sequences for qPCR are listed: *ATG5*: 5ʹ-3ʹ (sense) CACCTCTGCTTTCCTCCACT, 5ʹ-3ʹ (antisense) TAGGCCAAAGGTTTCAGCTTC; *ATG7*: 5ʹ-3ʹ (sense) GCCCCAGGAGATTCAACCA, 5ʹ-3ʹ (antisense) TAGCCCCCTTCTGGATGCT.

### RNA-binding protein immunoprecipitation (RIP)

SW1990 or PANC-1 cells were seeded into 10-cm dishes, and exposed to DMSO or THM for 24 h, then formaldehyde-crosslinked and sonicated in RIP lysis buffer (1% protease, 1% RNase inhibitor). Protein A/G magnetic beads were mixed with YTHDF2 antibody or IgG (control) for 1 h, then blocked with RIP wash buffer + tRNA (1 μg/mL) at 4 °C for 1 h. Once cell lysate centrifugation was complete, the supernatant was combined with the blocked beads at 4 ℃ for 1 h. The beads were de-crosslinked at 42 ℃ for 1 h, and the lysate was heated at 55℃. Ultimately, extracts were analyzed by RT-qPCR.

### *In vivo* assay

① For the subcutaneous xenograft model (BALB/c nude mice), approximately 1 × 10^7^ logarithmic-phase PANC-1 cells were injected subcutaneously into 4-week-old mice. Once tumors grew to approximately 15 mm^3^, different doses (60, 90 or 120 mg/kg) of THM or 0.9% saline solution received intraperitoneally every other day for 10 times. Tumor diameters were measured every other day using the formula V = 1/2 (width^2^ × length). After being sacrificed, the mice were weighed, and tumors and major organs (heart, lungs, liver, kidneys, and spleen) were fixed for histological examination, including H&E and IHC staining. Blood was also collected for biochemical analysis. All animal procedures were conducted ethically and approved (HSD-20230829-05) by the Animal Experimentation Center of Hangzhou Normal University (Hangzhou, China).

② In the PDX pancreatic cancer model, tumor samples were harvested from patients who had undergone resection for pancreatic ductal adenocarcinoma, cut into small sterile fragments, and subcutaneously implanted into 4-week-old BALB/c nude mice armpits. After the tumors had reached a certain size, the process was repeated using cannulated needles to allow for amplification. Once the tumor size reached approximately 15 mm^3^, THM was intraperitoneally injected every other day for 10 doses, while the GEM group received treatment every week. Tumor diameters were measured every other day, and volume was calculated as V = 1/2 (width^2^ × length). This experiment was approved (NO.2023-068) by the Ethical Review Committee of Zhejiang Provincial People's Hospital (Hangzhou, China).

③ In the orthotopic pancreatic cancer C57BL/6 model, 5×10⁶ Pan02-luc cells were injected into mice. After 4 days, mice were randomized into four groups: saline (vehicle), THM (90 mg/kg), αPD-1 (5 mg/kg), and αPD-1+THM. THM was administered every other day, and αPD-1 every 3 days. Tumor size was monitored *via* IVIS Lumina LT (Revvity, USA) and Biospace Photon Imager^TM^ Optima (Biospace Lab, France).

### Immunohistochemical (IHC) analysis

Fixed tumor tissues underwent paraffin embedding, sectioning, baking, dewaxing in xylene, and rehydration with gradient alcohol. Antigen retrieval was performed *via* high-temperature autoclave in 0.01 M citrate buffer. Sections were exposed to primary antibodies overnight, washed, and then treated with enzyme-labeled secondary antibodies for 1 h. Staining was performed with DAB and hematoxylin (Solarbio), and images were acquired using an Olympus VS200 scanner.

### Statistical analysis

Experiments were triplicated to ensure reliability, with results expressed as mean ± S.D. Statistical evaluation and visualization were conducted using GraphPad Prism 8.0, employing Two-Way ANOVA and student's t-test.

## Supplementary Material

Supplementary figures.
