# Supplementary material for: Tetrahydromagnolol induces autophagic cell death by targeting the m6A reader protein YTHDF2 and enhances the efficacy of anti-PD-1 immunotherapy in pancreatic cancer cells
Source: Theranostics. 2025 Jul 2;15(15):7726–46. doi: 10.7150/thno.112612 (PMC12316034; doi:10.7150/thno.112612)
Supplement: Supplementary file 1 — Supplementary figures. [file thnov15p7726s1.pdf]

Supplementary Information

Tetrahydromagnolol induces autophagic cell death by targeting the m^{6}A reader protein YTHDF2 and enhances the efficacy of anti-PD-1 immunotherapy in pancreatic cancer cells

Guohua Li[1, 2, #], Qibiao Wu[2, #], Yiping Mou[3, #] Yunhao Qiao[1, #], Lijun Jin[4], Qian Shi[1], Ruonan Zhang[1], Jie Li[1, 2], Yitian Sun[1, 2], Aili Zhang[1, 2], Haiyang Jiang[1], Zijing Yang[1], Zhiyu Zhu[1], Mengmeng Ma[1], Xiaoyu Sun[1], Xinbing Sui[1, 2, *]

[1] School of Pharmacy, Hangzhou Normal University, Hangzhou, 311121, Zhejiang, China

[2] State Key Laboratory of Quality Research in Chinese Medicines, Faculty of Chinese Medicine, Macau University of Science and Technology, Macau, P.R. China

[3]Department of Gastrointestinal and Pancreatic Surgery, Zhejiang Provincial People's Hospital, People's Hospital of Hangzhou Medical College, Hangzhou, Zhejiang, China

[4]Department of Traditional Chinese Medicine, The Second Affiliated Hospital, School of Medicine, Zhejiang University, Hangzhou, Zhejiang, China

*Correspondence: suilab@hznu.edu.cn (X.S.)

[#]These authors contribute equally.

Supplementary Figures:

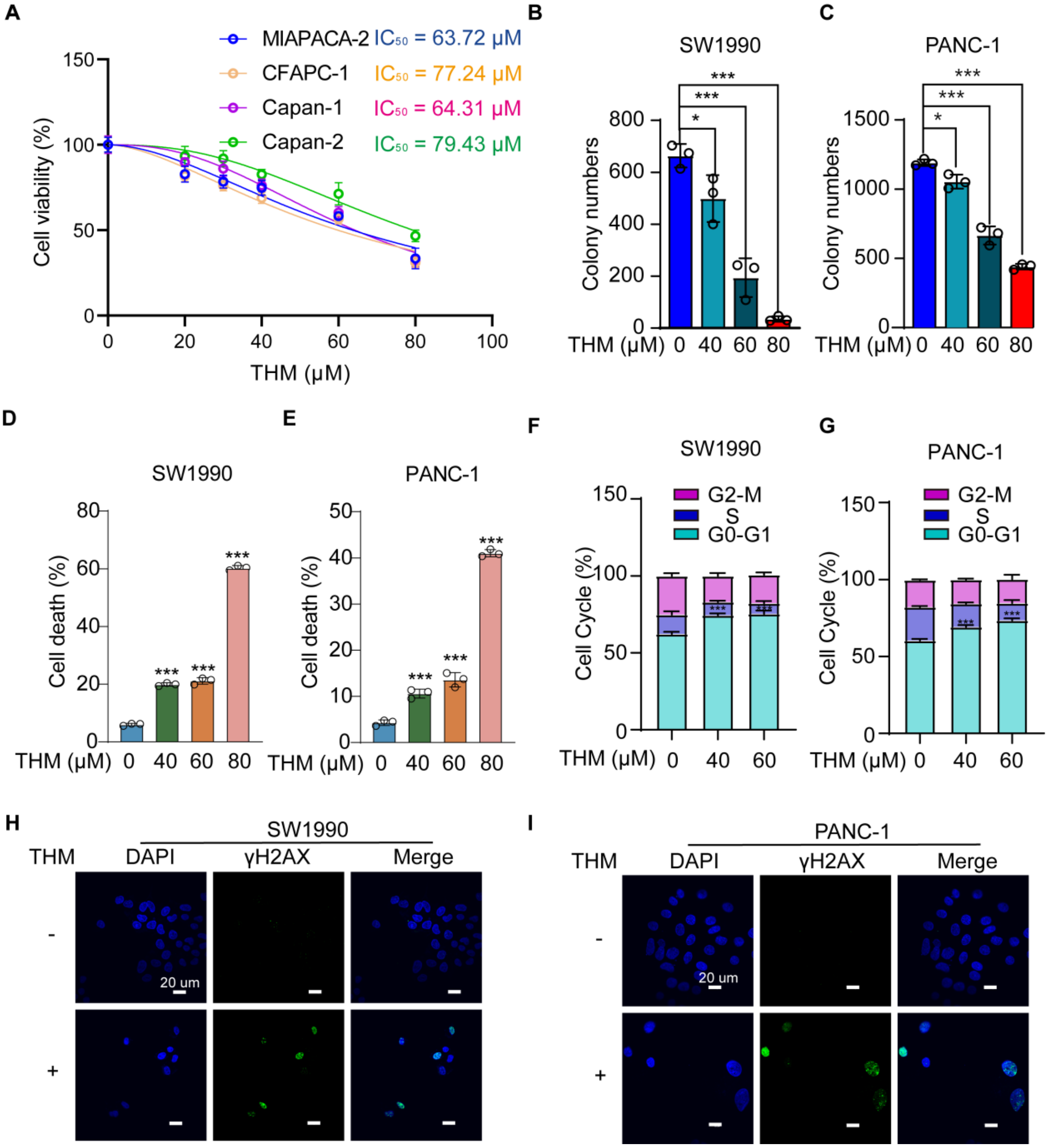

Figure S1. THM inhibited cell proliferation and induced cell death in pancreatic cancer cells. **(A)** MIAPaCa-2, CFAPC-1, Capan1 and Capan2 cells were treated with different concentrations of THM for 24 h, and cell viability was measured by CCK8 assay. **(B-C)** The quantitative analysis of the colony formation after the treatment with THM, means ±S.D., n = 3; *$p < 0.05$, ***$p < 0.001$. **(D-E)** The quantitative analysis of annexin V-FITC/ PI staining after the treatment with THM for 24 h, means ±S.D., n = 3; **$p < 0.01$, ***$p < 0.001$. **(F-G)** The quantitative analysis of cell cycle after the treatment with THM for 24 h. **(H-I)** Immunofluorescence staining for DNA damage marker γ-H2AX in SW1990 and PANC-1 cells after THM treatment for 24 h.

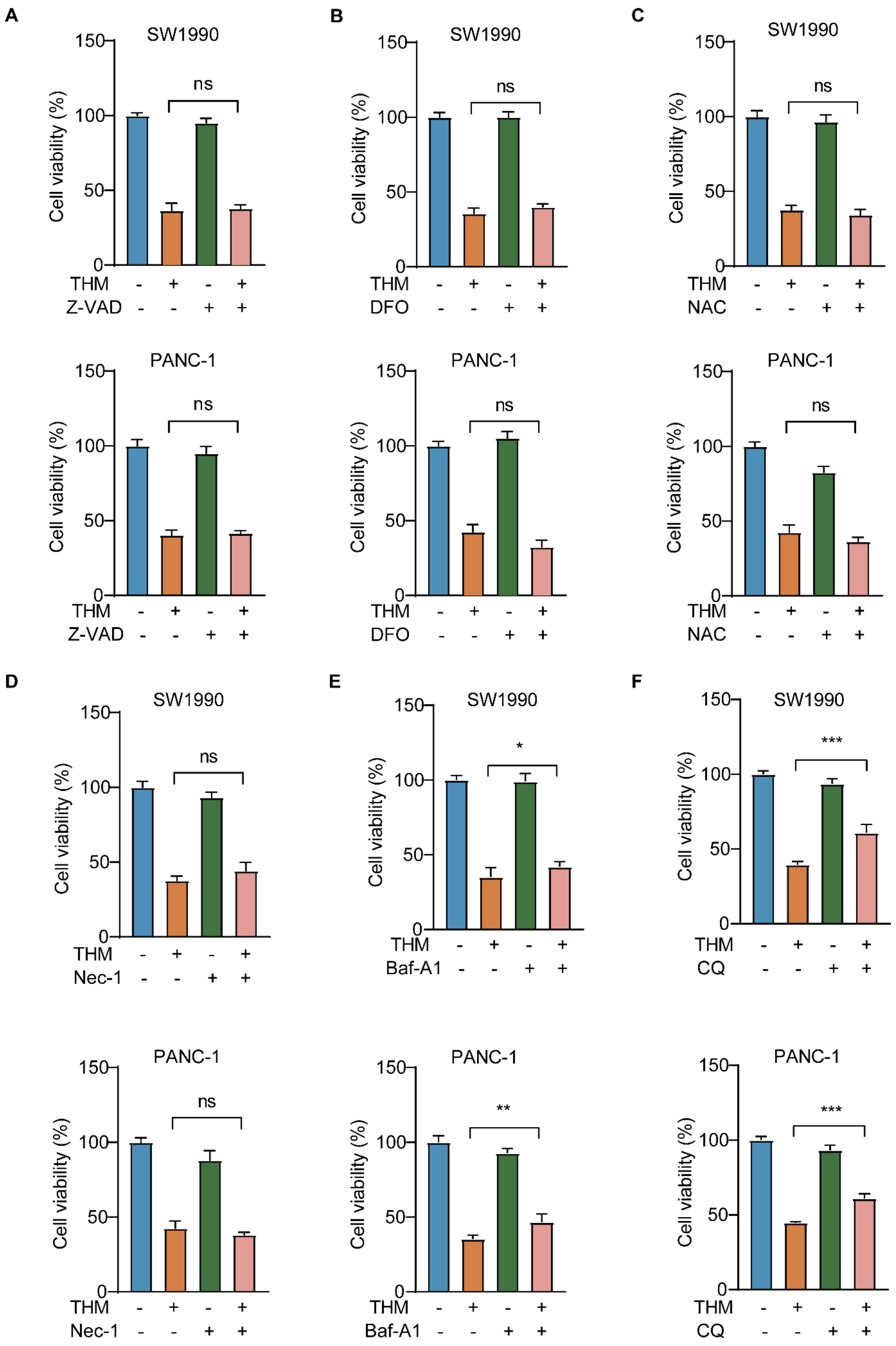
A
SW1990
Cell viability (%)
150
100
50
0
ns
THM - + - +
Z-VAD - - + +
PANC-1
Cell viability (%)
150
100
50
0
ns
THM - + - +
Z-VAD - - + +
B
SW1990
Cell viability (%)
150
100
50
0
ns
THM - + - +
DFO - - + +
PANC-1
Cell viability (%)
150
100
50
0
ns
THM - + - +
DFO - - + +
C
SW1990
Cell viability (%)
150
100
50
0
ns
THM - + - +
NAC - - + +
PANC-1
Cell viability (%)
150
100
50
0
ns
THM - + - +
NAC - - + +
D
SW1990
Cell viability (%)
150
100
50
0
ns
THM - + - +
Nec-1 - - + +
PANC-1
Cell viability (%)
150
100
50
0
ns
THM - + - +
Nec-1 - - + +
E
SW1990
Cell viability (%)
150
100
50
0
*
THM - + - +
Baf-A1 - - + +
PANC-1
Cell viability (%)
150
100
50
0
**
THM - + - +
Baf-A1 - - + +
F
SW1990
Cell viability (%)
150
100
50
0
***
THM - + - +
CQ - - + +
PANC-1
Cell viability (%)
150
100
50
0
***
THM - + - +
CQ - - + +

Figure S2. The effect of THM alone or combines with different cell death inhibitors on the cell viability of pancreatic cancer cells. (**A**) SW1990 and PANC-1 cells were treated with THM with or without Z-VAD (10 μM) for 24 h, and cell viability was detected. (**B**) SW1990 and PANC-1 cells were treated with THM with or without DFO (10 μM) for 24 h, and cell viability was detected. (**C**) SW1990 and PANC-1 cells were treated with THM with or without NAC (1 mM) for 24 h, and cell viability was detected. (**D**) SW1990 and PANC-1 cells were treated with THM with or without Nec-1 (10 μM) for 24 h, and cell viability was detected. (**E**) SW1990 and PANC-1 cells were treated with THM with or without Baf-A1 (20 nM) for 24 h, and cell viability was detected. (**F**) SW1990 and PANC-1 cells were treated with THM with or without CQ (25 μM) for 24 h, and cell viability was detected. Data are summarized as mean ± S.D., *$p < 0.05$, **$p < 0.01$, ***$p < 0.001$, ns: not significant (n = 3).

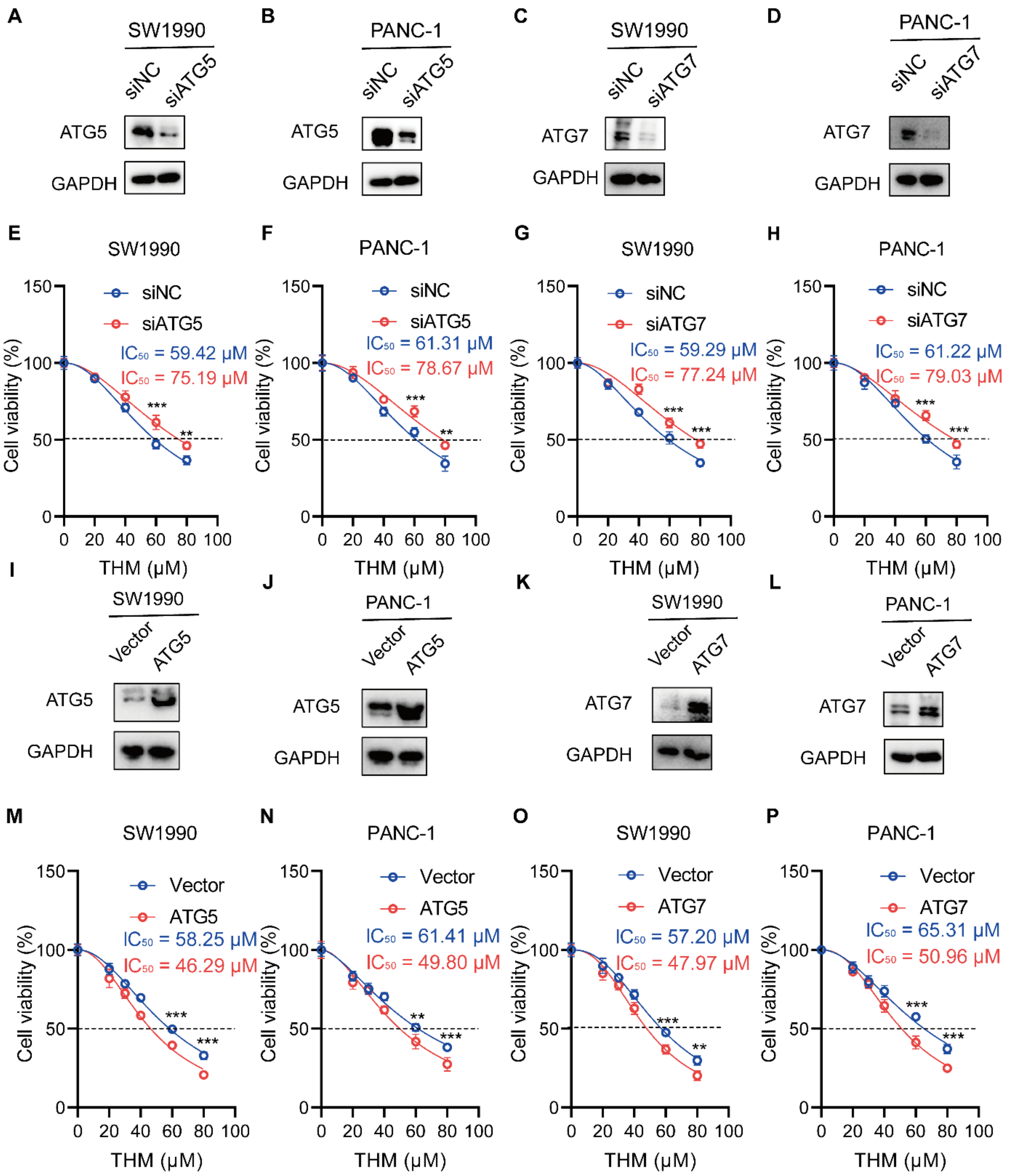

Figure S3. THM affected the cytotoxicity of pancreatic cancer cells through autophagy-related proteins ATG5 and ATG7 **(A-D)** ATG5/7 proteins in knocked-down SW1990 and PANC-1 cells were analyzed by western blot. GAPDH was used as internal control. **(E-H)** SW1990 and PANC-1 cells were treated with different concentrations of THM for 24 h after knocking down the expression of ATG5/7, and cell viability was measured by CCK8 assay. n = 3; $^{**}p < 0.01$, $^{***}p < 0.001$. **(I-L)** ATG5/7 proteins in over-expressed SW1990 and PANC-1 cells were analyzed by western blot. GAPDH was used as internal control. **(M-P)** SW1990 and PANC-1 cells were treated with different concentrations of THM for 24 h after overexpression of ATG5/7, and cell viability was measured by CCK8 assay. n = 3; $^{**}p < 0.01$, $^{***}p < 0.001$.

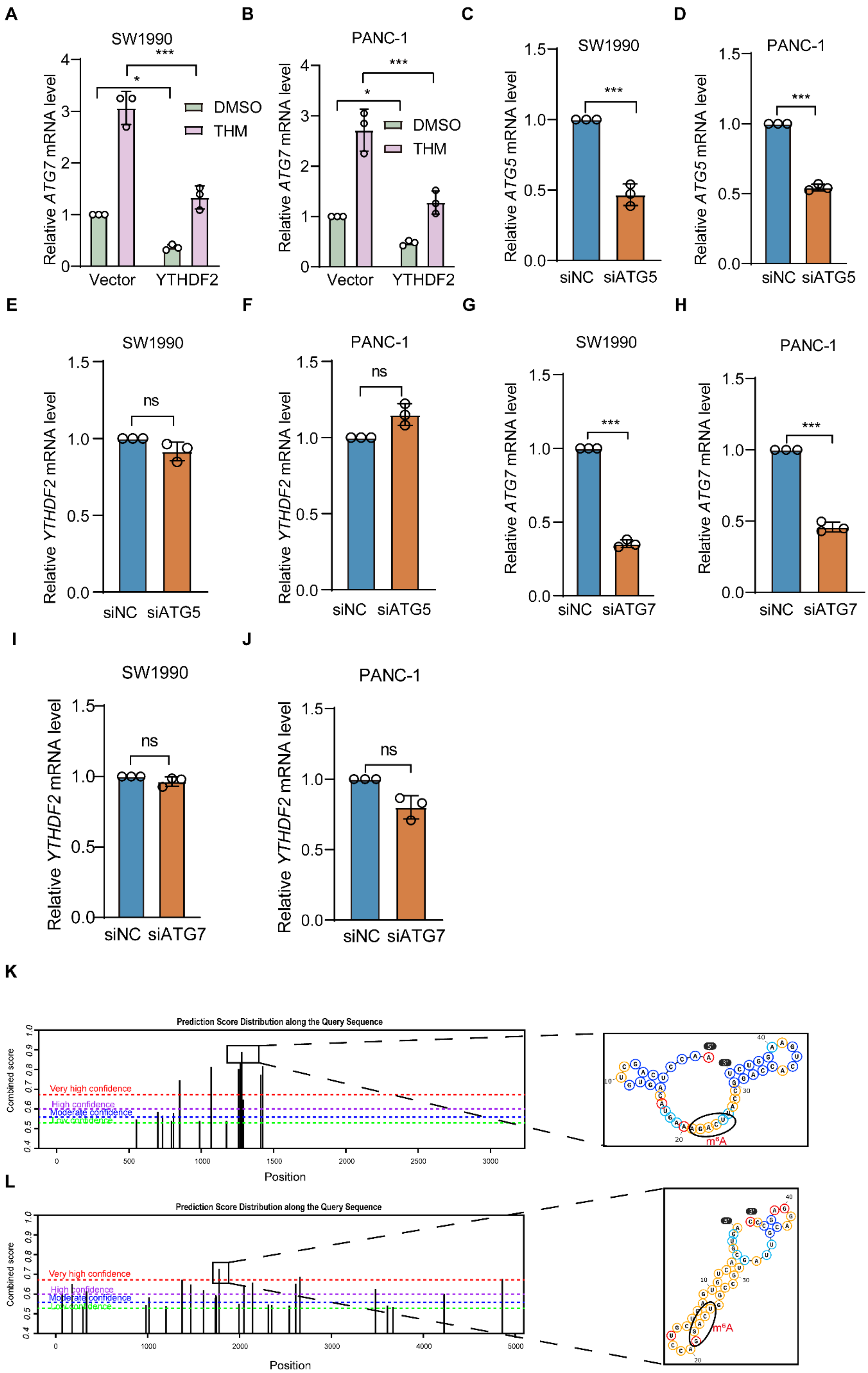

A
SW1990
Relative ATG7 mRNA level
DMSO
THM
Vector
YTHDF2
B
PANC-1
Relative ATG7 mRNA level
DMSO
THM
Vector
YTHDF2
C
SW1990
Relative ATG5 mRNA level
siNC
siATG5
D
PANC-1
Relative ATG5 mRNA level
siNC
siATG5
E
SW1990
ns
Relative YTHDF2 mRNA level
siNC
siATG5
F
PANC-1
ns
Relative YTHDF2 mRNA level
siNC
siATG5
G
SW1990
Relative ATG7 mRNA level
siNC
siATG7
H
PANC-1
Relative ATG7 mRNA level
siNC
siATG7
I
SW1990
ns
Relative YTHDF2 mRNA level
siNC
siATG7
J
PANC-1
ns
Relative YTHDF2 mRNA level
siNC
siATG7
K
Prediction Score Distribution along the Query Sequence
Combined score
Very high confidence
High confidence
Moderate confidence
Low confidence
Position
m6A
L
Prediction Score Distribution along the Query Sequence
Combined score
Very high confidence
High confidence
Moderate confidence
Low confidence
Position
m6A

Figure S4. THM suppressed YTHDF2 recognition of m^{6}A mRNA targets. **(A-B)** Analysis of *ATG7* mRNA expression in SW1990 and PANC-1 cells by RT-qPCR after overexpression of YTHDF2 with or without THM treatment. n = 3; ***p < 0.001. **(C-F)** Analysis of *ATG5* and *YTHDF2* mRNA expression in SW1990 and PANC-1 cells by RT-qPCR after knockdown of ATG5. n = 3; **p < 0.01. **(G-J)** Analysis of *ATG7* and *YTHDF2* mRNA expression in SW1990 and PANC-1 cells by RT-qPCR after knockdown of ATG7. n = 3; **p < 0.01. **(K-L)** The predicted m^{6}A sites of *ATG5* and *ATG7* mRNA via SRAMP (http://www.cuilab.cn/sramp/).

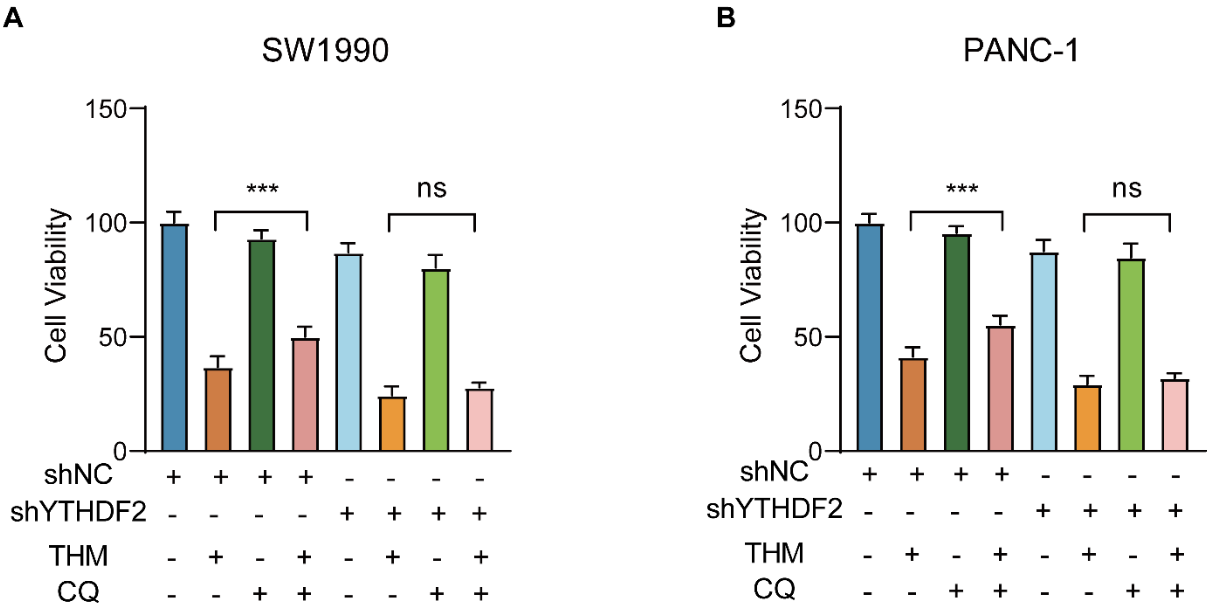

Figure S5. Effects of THM on cell viability through YTHDF2 and autophagy regulation in pancreatic cancer cells. (**A-B**) Analysis of cell viability in SW1990 and PANC-1 cells by CCK8 assay after transfection with shNC or shYTHDF2 and treatment with THM with or without CQ for 24 h. Data are presented as mean ± S.D. of three independent experiments. ***p < 0.001, ns: not significant.

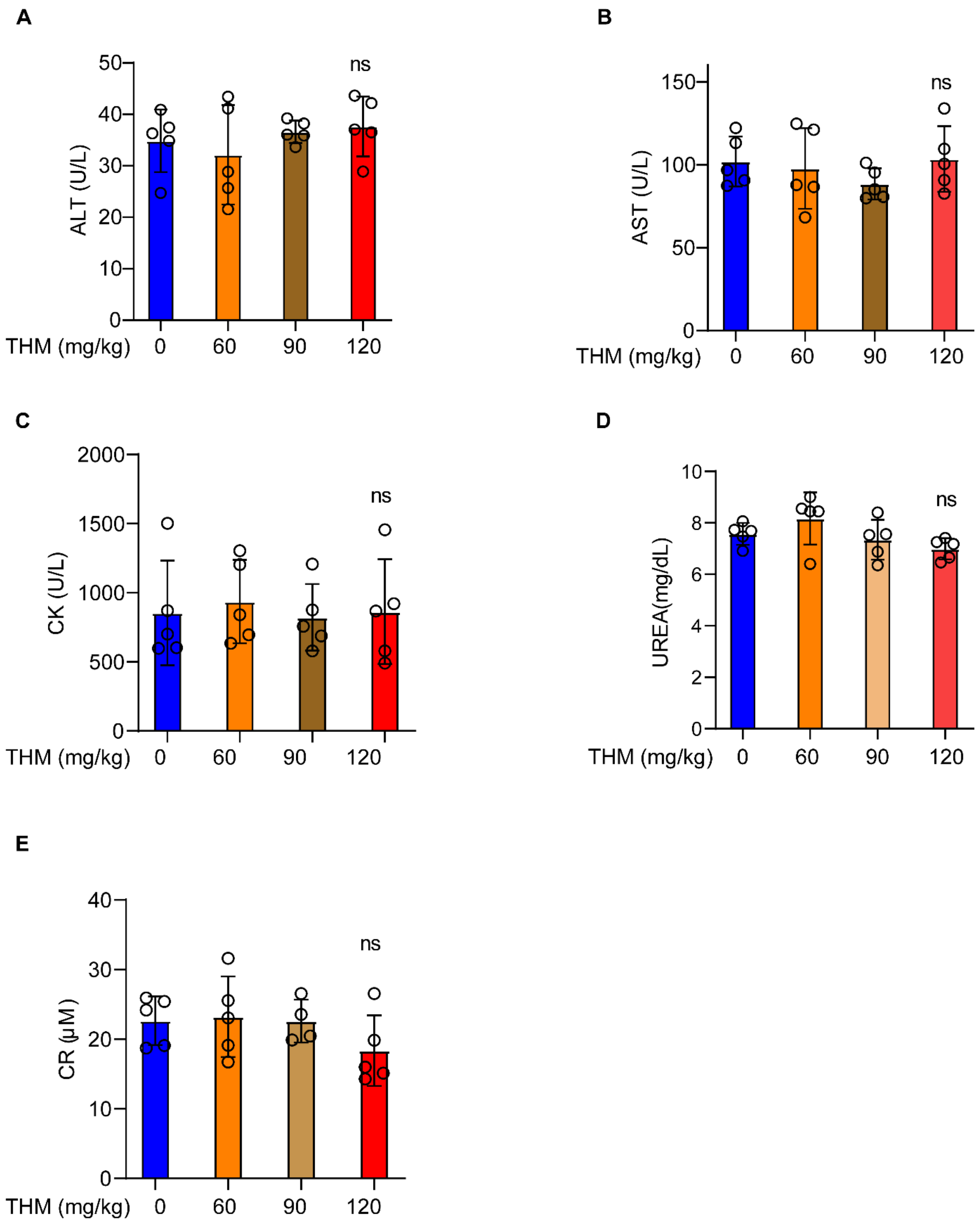

Figure S6. Safety evaluation of THM on pancreatic cancer was evaluated in xenograft models *in vivo*. **(A)** After treatment with saline or THM for 10 times, mice serum was taken for the determination of alanine transaminase (ALT) assay. **(B)** After treatment with saline or THM for 10 times, mice serum was taken for aspartate aminotransferase (AST) assay. **(C)** After treatment with saline or THM for 10 times, mice serum was taken for creatine kinase (CK) assay. **(D)** After treatment with saline or THM for 10 times, mice serum was taken for urea (UREA) assay. **(E)** After treatment with saline or THM for 10 times, mice serum was taken for

creatinine (CR) assay. All the data are presented as means ±S.D. ns: there was no significant difference.

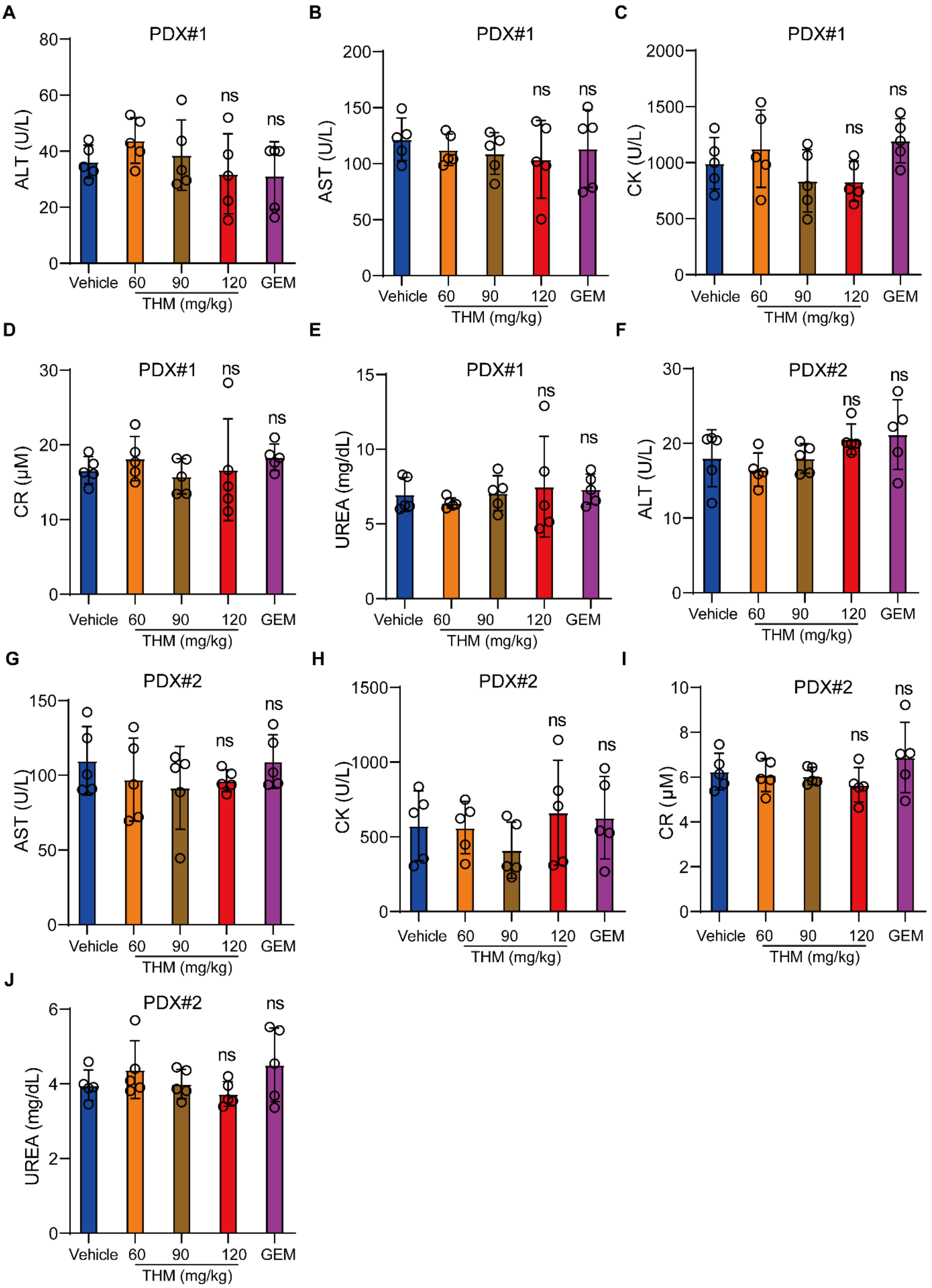

Figure S7. Safety evaluation of THM on pancreatic cancer was evaluated in PDX models *in vivo*. **(A)** PDX#1 mice serum was taken for the determination of ALT assay in different groups. **(B)** PDX#1 mice serum was taken for AST assay in different groups. **(C)** PDX#1 mice serum

was taken for CK assay in different groups. **(D)** PDX#1 mice serum was taken for CR assay in different groups. **(E)** PDX#1 mice serum was taken for UREA assay in different groups. **(F)** PDX#2 mice serum was taken for the determination of ALT assay in different groups. **(G)** PDX#2 mice serum was taken for aspartate aminotransferase (AST) assay in different groups. **(H)** PDX#2 mice serum was taken for CK assay in different groups. **(I)** PDX#2 mice serum was taken for CR assay in different groups. **(J)** PDX#2 mice serum was taken for UREA assay in different groups. All the data are presented as means ±S.D. ns: there was no significant difference.

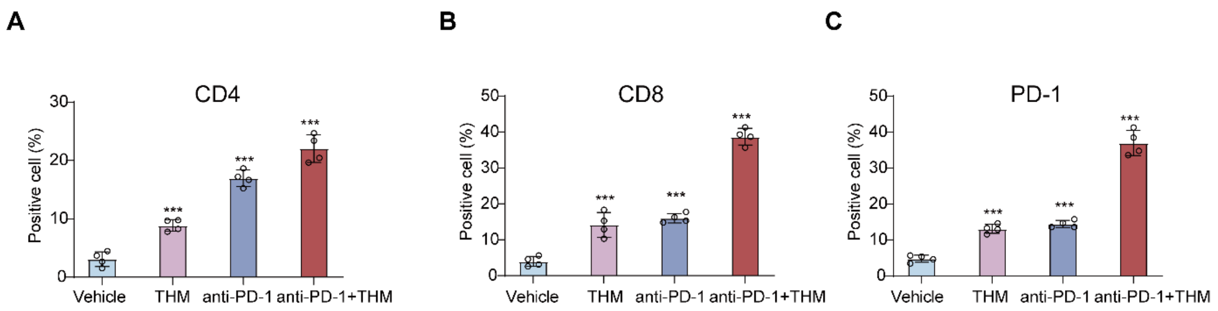

Figure S8. Effects of THM and anti-PD-1 combination therapy on immune infiltration in pancreatic cancer. **(A-C)** Quantitative analysis of immunohistochemical staining for CD4, CD8a and PD-1 in orthotopic Pan02-Luc pancreatic cancer tumor sections. Data are summarized as mean ± S.D., ****p* < 0.001, ns: not significant (n = 4).

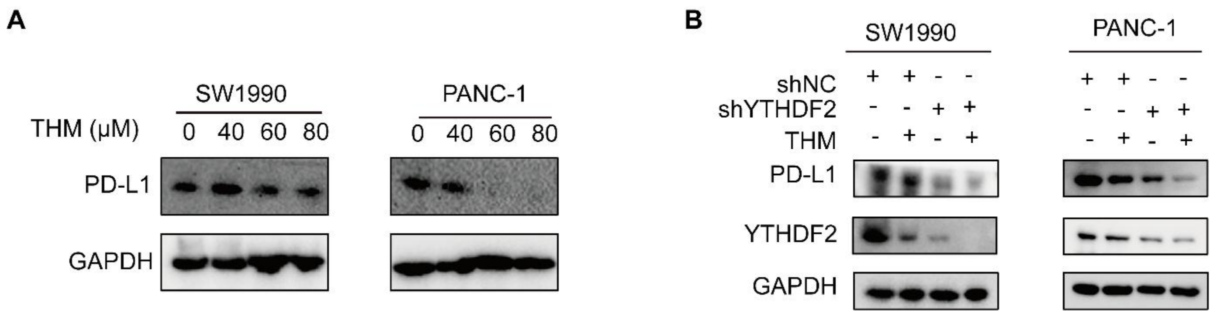

Figure S9. Effects of THM on PD-L1 expression in pancreatic cancer cells *via* the YTHDF2 signaling pathway. **(A)** SW1990 and PANC-1 cells were treated with different concentrations of THM for 24 h, and PD-L1 protein levels were detected by western blot. **(B)** SW1990 and PANC-1 cells with stable shNC or shYTHDF2 were treated with THM for 24 h, and PD-L1 and YTHDF2 protein levels were detected by western blot.
